# Ubiquitous Carbohydrate Binding Modules Decorate 936 Lactococcal Siphophage Virions

**DOI:** 10.3390/v11070631

**Published:** 2019-07-09

**Authors:** Stephen Hayes, Jennifer Mahony, Renaud Vincentelli, Laurie Ramond, Arjen Nauta, Douwe van Sinderen, Christian Cambillau

**Affiliations:** 1 School of Microbiology, University College Cork, T12 YT20 Cork, Ireland; 2 Architecture et Fonction des Macromolécules Biologiques, Aix-Marseille Université, Campus de Luminy, 12388 Marseille, France; 3 Architecture et Fonction des Macromolécules Biologiques, Centre National de la Recherche Scientifique (CNRS), Campus de Luminy, 13288 Marseille, France; 4 FrieslandCampina, 3800 Amersfoort, The Netherlands; 5 APC Microbiome Ireland, University College Cork, T12 YT20 Cork, Ireland

**Keywords:** bacteriophage, *Lactococcus lactis*, receptor-binding protein, carbohydrate binding module, phage–host interactions

## Abstract

With the availability of an increasing number of 3D structures of bacteriophage components, combined with powerful in silico predictive tools, it has become possible to decipher the structural assembly and functionality of phage adhesion devices. In the current study, we examined 113 members of the 936 group of lactococcal siphophages, and identified a number of Carbohydrate Binding Modules (CBMs) in the neck passage structure and major tail protein, on top of evolved Dit proteins, as recently reported by us. The binding ability of such CBM-containing proteins was assessed through the construction of green fluorescent protein fusion proteins and subsequent binding assays. Two CBMs, one from the phage tail and another from the neck, demonstrated definite binding to their phage-specific host. Bioinformatic analysis of the structural proteins of 936 phages reveals that they incorporate binding modules which exhibit structural homology to those found in other lactococcal phage groups and beyond, indicating that phages utilize common structural “bricks” to enhance host binding capabilities. The omnipresence of CBMs in Siphophages supports their beneficial role in the infection process, as they can be combined in various ways to form appendages with different shapes and functionalities, ensuring their success in host detection in their respective ecological niches.

## 1. Introduction

Bacteriophages (or phages) of *Lactococcus lactis* are well known to cause fermentation disruptions and product inconsistencies with a consequent economic cost to the dairy industry [1], which in turn has fuelled intense scientific scrutiny of such phage groups [2]. Chief among the most commonly encountered lactococcal phages are those belonging to the lytic 936 group [3], for which 164 complete genome sequences are now publicly available (NCBI Database). The availability of such a large number of phage-group specific genome sequences is rather unique, and renders the 936 group an ideal model group for the study of phage–host interactions.

An initial, yet crucial step in the phage infection process is the specific recognition of, and binding to the bacterial host. Tailed phages of the order *Caudovirales*, including the lactococcal 936 group phages, recognize and bind to their host utilizing a complex adhesion device at the distal end of their tail, which has been referred to as the baseplate [4]. The distinct baseplate structures of lactococcal phages TP901-1 (which belongs to the so-called P335 group) [5,6,7] and p2 (a 936 group phage) [8] have previously been resolved. The baseplate of p2 is a complex assembly of three proteins—the Dit (Distal tail protein), the Tal (Tail associated lysin), and the RBP (Receptor Binding Protein) [8]. The ~1 MDa complex was deduced to consist of a central Dit hexameric ring, with a trimer of RBP proteins attached to each Dit. A Tal protein trimer ‘plugs’ the central cavity of the Dit ring, and a second Dit hexameric ring, back-to-back with the first, completes the baseplate complex. Crystallization in both the absence and presence of Sr^++^ (a cationic substitute for Ca^++^) revealed that the p2 baseplate possesses two possible conformations; the “closed” state in the absence of Ca^++^/Sr^++^, with the RBPs oriented upwards in the “heads-up” conformation, which is incompatible with host binding; and the “open” state following activation by the presence of Ca^++^/Sr^++^, with the RBPs rotated 200° downwards in the “heads-down” conformation, thereby allowing host binding [8]. This hetero-proteinaceous assembly and associated structure is highly conserved amongst 936 group phages, although in three phages a variation has been observed regarding the incorporation of two different RBP types [9].

The structural and functional characteristics of the RBPs of two members (p2 and bIL170) of the 936 phage group have been studied in detail [10,11,12], which allowed the identification of conserved and distinctive features. One of the key findings of these studies has been the discovery of a conserved modularity between 936 group RBPs, with all exhibiting so-called head, neck, and shoulder domains in their structures [13]. The shoulder domain of the p2 RBP attaches the RBP to the Dit, and is comprised of a β-sandwich fold assembling two 4-stranded anti-parallel β-sheets [10], with a long helical domain in each RBP allowing it to associate in trimeric form with two additional RBPs. The neck domain is formed by a triple-stranded β-helix organized into four β-strands, resulting in a rigid structure. The RBP head domain forms a β-barrel comprised of seven anti-parallel β-strands, and incorporates a carbohydrate binding site to facilitate phage–host binding [11]. This binding site is thought to recognize the Cell Wall PolySaccharide (CWPS) receptor on the surface of the lactococcal cell wall. These polysaccharides are encoded by the large *cwps* gene cluster, which comprises a highly conserved 5′ region and a 3′ region of genetic diversity. Comparative analysis of these diverse regions allowed the classification of lactococcal strains into at least three different CWPS types based on the genetic composition of the *cwps* gene cluster: CWPS type A, e.g., *L. lactis* UC509.9 and CV56, CWPS type B, e.g., *L. lactis* IL1403 and KF147, and CWPS type C, e.g., *L. lactis* MG1363 and SK11 [14]. Furthermore, it has been established that C type CWPS strains can be further divided into five subtypes (C_1_–C_5_) based on a variable region encoding glycosyltransferases in these strains [15]. Previous studies have demonstrated that 936 group phage RBPs can be divided into at least five groups based on nucleotide sequence comparisons and corresponding host CWPS type [14,16].

It was believed that the RBP was the sole facilitator of host binding in 936 group phages. However, it has recently been demonstrated that a large number of 936 phages employ a so-called “evolved” Dit protein [17]. These elongated Dits possess an internal insertion which exhibits structural homology to the BppA (accessory baseplate protein) Carbohydrate Binding Module (CBM), itself an ancillary baseplate protein in the P335 phage Tuc2009 distinct from the RBP, but involved in host binding [18,19].

Here, we further explored the same 936 phage group used in the above-mentioned evolved Dit study. Firstly, by performing HHpred analysis of the head region of the RBPs, we determined that not only can 936 phage RBPs be grouped based on their predicted structure, but that these phages incorporate RBP structures that are similar to a variety of non-936 group lactococcal phages. Similar to previous groupings of 936 RBPs, these groupings correspond to the CWPS type of their host. We then set out to identify other host-recognition options beyond evolved Dit CBMs, that these virions may have adopted by incorporating CBMs in their tail and neck structural proteins. Using HHpred analysis and subsequent experimental verification, we determined that the vast majority of 936 phages indeed incorporate such additional CBMs in their tail and neck, and that these CBMs are involved in adhesion to the host in addition to the activities of the RBP. The stacking of multiple CBMs in 936 group phages allows the assembly of a variety of structures using the same pool of building blocks. Simultaneously, this approach is ideal for the identification of domains of currently unknown function/structure so as to facilitate a next generation of structure-function studies to advance current knowledge of phage–host interactions of the ubiquitous *Siphoviridae* phages.

## 2. Materials and Methods

### 2.1. Hosts and Bacteriophages

The accession numbers of the phages examined in this study can be found in Supplementary Table S1. A total of 45 strains of the bacterial species *L. lactis* were used in this study (Supplementary Table S2). Bacterial strains were cultured overnight at 30 °C in M17 broth (Oxoid, Hampshire, UK) supplemented with either 0.5% *w*/*v* lactose (LM17) in the case of strains A-T and 1-20, or 0.5% *w*/*v* glucose (GM17) for all other strains.

### 2.2. Host CWPS Genotyping

In order to perform a thorough investigation of the previously reported correlation between phage RBP group and host CWPS type [16], two CWPS typing multiplex PCRs were performed on 40 host strains previously isolated from a Dutch dairy facility [20]. The first, which classifies hosts as belonging to the A, B, or C type based on the unique genomic regions associated with each CWPS type, was performed as previously described [14]. For those strains which were classified as a C-type, a second multiplex PCR was performed which classifies C-type strains into subtypes C_1_–C_5_ [15,21]. Primers and expected amplicon sizes are presented in Supplementary Table S3.

### 2.3. Phage Host-Range Assays

To investigate the link between phage RBP(-head) groups and corresponding host CWPS types, phage host range assays were performed using 62 phages that had previously been isolated from Dutch dairy facilities [16,17,20] against the 40 strains whose CWPS types had been determined. These host-range surveys were performed using the double-layer spot assay method [22]. For this purpose, 5 µL of each phage lysate (titres between 10^7^ and 10^8^ PFU/mL) was spotted on an overlay of the relevant host strain. The host range of each phage was determined as those bacterial hosts against which spots were formed.

### 2.4. Bioinformatic Analysis

The protein sequence and structure analysis was performed using HHpred (Homology detection & structure prediction by HMM-HMM comparison) using the default settings [23,24]. The sequence alignments were performed with Multalin [25]. Modular visualization and modelling were performed with Coot [26,27], Chimera [28] and Pymol [29].

### 2.5. CBM-Encoding Gene Cloning

Sequences encoding the putative carbohydrate binding domains from the TpeX of Phi2R06A and the Neck Passage Protein (NPS) CBMs (NPS-CBMs) of PhiA.16 were amplified for cloning and expression in *Escherichia coli* (Dataset S1). Phage DNA was isolated using the Norgen Biotek Phage DNA Isolation Kit (Ontario, Canada), and the regions of interest were amplified by PCR (KOD Hot Start Polymerase, Merck Millipore, Cork, Ireland). Purified PCR products were cloned into pHTP9 (Green Fluoresceni Protein, GFP) using the NZYEasy Cloning Kit (NZYTech genes & enzymes, Lisbon, Portugal). The recombinant proteins possessed a hexa-histidine tag for purification, as well as a TEV recognition sequence. Recombinant plasmids were transformed using a high-throughput method into NZY5α competent *E. coli* as previously described [30].

### 2.6. Protein Production

The two recombinant plasmids were used to transform Rosetta™ (DE3) pLysS *E. coli* cells. For recombinant protein production, 100 mL of auto-induction medium containing kanamycin (50 μg/mL) and chloramphenicol (34 μg/mL) was inoculated (1/100 *v*/*v*) for each protein. Cultures were then incubated for 24 h at 25 °C in a Multitron Standard shaking incubator (INFORS-HT, Switzerland) (300 rpm). Cells were collected by centrifugation and resuspended in a volume of lysis buffer dependent on the final culture OD of the individual expression, which was then frozen at −80 °C overnight. Cells were thawed, and incubated with 10 µg/mL DNAse and 20 mM MgSO_4_ for 30 min shaking at 24 °C. To ensure complete cell lysis, cells were sonicated (Soniprep 150, MSE, UK) for 5 min (power 15, 30 s ON/OFF cycles), and subsequently centrifuged for 30 min at 20,000× *g*. Proteins were then purified using Ni Sepharose 6 Fast Flow resin (GE Healthcare, Uppsala, Sweden) and eluted with 250 mM imidazole elution buffer (50 mM Tris, 300 mM NaCl, 250 mM imidazole, pH 8.0). Purified protein was dialyzed overnight against protein buffer (50 mM Tris, 300 mM NaCl, pH 8.0) in Thermo Scientific™ Slide-A-Lyzer™ 10K MWCO dialysis cups, prior to storage at −80 °C until further use.

### 2.7. Fluorescent Binding Assays

Cell binding assays using fluorescently labelled TpeX and NPS proteins were performed as described previously [31], with a number of modifications. Briefly, 0.3 mL of the relevant host cells (Figure 3) in the exponential growth phase were harvested and resuspended in 100 µl of SM buffer (50 mM Tris-HCl pH 7.5, 100 mM NaCl, 10 mM MgSO_4_). Cells were incubated with between 5 µg and 100 µg (as appropriate) of fluorescently labelled protein for 12.5 min at 37 °C. Cells were washed twice in SM buffer, and fluorescent binding was visualized by microscopy. Initial fluorescent binding assays were viewed via fluorescent microscopy at a magnification of 60× (Olympus AX70 Provis Upright Research Microscope, Olympus Corporation, Japan). To achieve high-resolution images, fluorescent binding of these proteins was then visualized via confocal microscopy (Zeiss LSM 5 Exciter, Zeiss, Germany) with a wavelength of 488 nm for GFP excitation. Images were analyzed and compiled using the Zen 2.3 Lite software package (Zeiss, Germany).

## 3. Results

### 3.1. Targets of the Bioinformatic Analyses

The 936 phage group represents an industrially relevant group of phages that has been thoroughly studied in recent years [3]. The structure of the entire virion of the 936 phage p2 has been resolved by electron microscopy (EM) [32], while the RBP structures of phages p2 and bIL170 have been determined by X-ray crystallography [5,10,12], providing a highly useful resource for the analysis of structural components of these phages. However, as more genome sequences become available, it is apparent that various phage–host interactions may be in operation. Indeed, in a recent comprehensive analysis of the Dit proteins of 113 members of the 936 group, it was revealed that many incorporate an internal extension encoding a BppA-like CBM, which is involved in phage–host interactions [17]. In the current work, we aimed to expand upon this study and determine whether similar adhesion modules are present in other 936 phage structural components using the same set of 113 phages, and if, like the “evolved” Dits, these share structural similarity to those present in other genetically and structurally analyzed phages. The successful identification of the BppA-like domain in the “evolved” Dits in the previous study also prompted a similar examination of the RBPs of the 936 group, to determine if conserved structural motifs could be identified.

**Figure 1:**
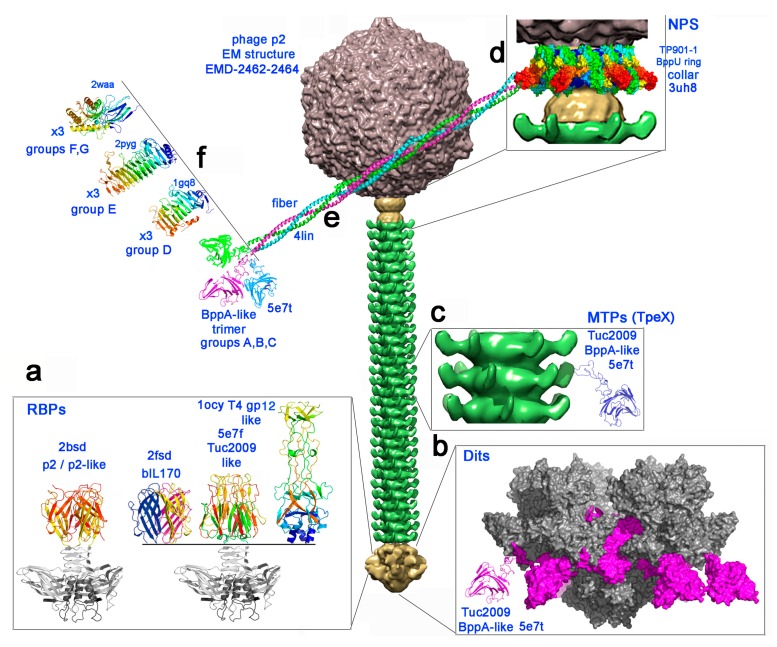
The carbohydrate binding modules of 936 group phages represented on the electron microscopy structure of phage p2 [32]. From top to bottom, the capsid (grey), the connector (yellow), the tail (green) and the baseplate (yellow). (**a**) The four types of receptor binding protein head domains [10] found in the 936 family. The constant shoulders and neck domain is depicted in grey, and the head domains are rainbow colored. (**b**) A structural model (molecular surface) of the previously described [9] BppA insertion (violet) within the evolved Dit arm from a 936 phage baseplate (grey) [8]. (**c**) The TpeX BppA-containing extension is represented in ribbon mode in blue, inserted after the protruding Major Tail Protein (MTP) adhesin domain. (**d**–**f**) The model of the neck passage proteins with the collar surrounding the connector (**d**, molecular surface), the triple helix bundle of the six fibers (**e**, ribbon mode), terminated by one of the four possible trimeric carbohydrate binding modules (CBM) domains (**f**, ribbon mode).

From the available genome sequences of the 936 phage group, a number of ORFs that encode products corresponding to a number of structural proteins in the phage tail were selected. These components were the Receptor Binding Protein (RBP), the Major Tail Protein (MTP), and the so-called Neck Passage Structure (NPS), (Dit proteins have previously been investigated and excluded from this study [17]). MTPs were analyzed during previous EM characterization of the p2 virion structure [32] revealing the presence of adhesin-associated decorations along the tail due to an MTP extension of its core module. Even longer MTP extensions, which result from a programmed translational frameshift encoding the so-called tail protein extension (TpeX), have recently been identified for certain 936 family members [16], with a role in phage–host interactions hypothesized [9]. The NPS ORFs that are present in certain 936 phages were also included in the current analysis as these appear to represent non-essential structural components with a putative involvement in host recognition [33]. For these various proteins of interest, corresponding amino acid sequences were extracted from 113 publicly available 936 group genomes, and sequence alignments were created. These alignments revealed distinct groups for each protein, and representative members from each group were then selected for HHpred analysis to identify regions of structural homology and putative CBM domains. The output of this HHpred analysis identified a number of CBMs throughout the structure of 936 group phages (Table 1 and Supplementary Table S1), and furthermore, identified structural homology between these CBMs and those of other non-936, and non-lactococcal phages (Figure 1).

### 3.2. Bioinformatic Analysis of Receptor Binding Proteins (RBPs)

**Figure 2:**
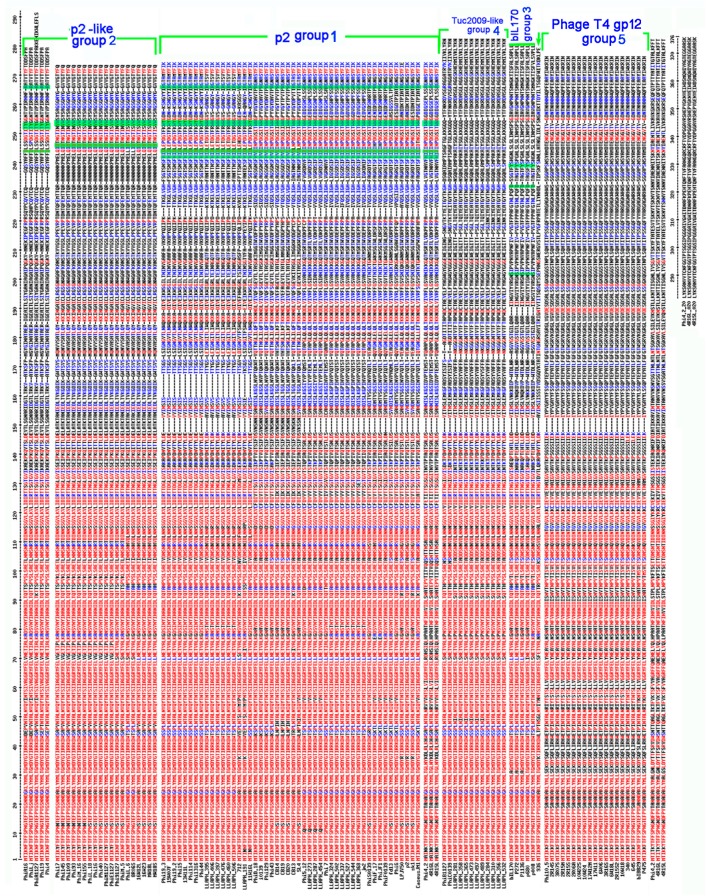
Sequence alignment of the RBP proteins. The various RBP groups, based on the predicted structure of the RBP head domain, are identified on the right of the alignment. The conserved amino acids are in red and the partially conserved in blue. Otherwise in black. The phage p2 4 essential amino-acids for receptor binding, Trp144, His232, Asp234 and Arg256, are conserved in the p2 class and only partly in the p2-like class. They are highlighted in green. This is not a cited image. Multalin is a program Performed with Multalin.

All RBPs of the 936 group exhibit very similar sequences within their first 135 *N*-terminal residues, corresponding to the shoulder and neck domains identified in the phage p2 RBP structure [10] (Figure 2). No insertions or deletions were observed in this region of the RBPs, indicating that the core domain and loops have conserved structures (Figure 1a grey domains, Figure 2). Beyond residue 135, however, both the sequence and length of the head domain vary considerably, and a number of ‘RBP-head’ groups can be discerned (Figure 2). Despite the presence of a number of deletions/insertions at the beginning of the head domain, the largest and most conserved group is that of phage p2. An important characteristic linking the members of this RBP-head group is the presence of a number of conserved amino acids within the sugar binding pocket previously identified in the phage p2 RBP head, represented by an aromatic residue (Trp 144), and Arg 256, Asp 234 and His 232 [10,11].

The second largest RBP-head group is typified by sequence divergence from phage p2 and binding site residues that are not conserved. This group is termed the p2-like group (Figure 2). HHpred analysis detected structural similarity between the head domains of all members of this group (100% probability of structural similarity to p2 head domain PDB ID:1ZRU/2BSE), and sequence alignments were consistent with this prediction (Figure 2). Examination of their sugar binding domain and surrounding sequences indicates that certain residues may be part of a binding site which is partially similar to that present in the RBP of phage p2 (Figure 2; green surligned residues). A subset of this group does not possess the conserved His (upper part of the p2-like group in Figure 2), while another subset of this group does not possess the conserved Arg (lower part of this group in Figure 2).

The third RBP-head group, the so-called bIL170 group, is represented by five phages, including bIL170 (Figure 2), for which the RBP head structure had previously been reported (PDB 2FSD) [12]. The bIL170 RBP head exhibits a similar yet distinct fold to that of phage p2. A putative saccharide binding site of phage bIL170 was proposed to involve Tyr 226, Asp 220 and Ser 191, which are fully conserved among members of this group.

The fourth, and so-called ‘Tuc2009-like’ RBP-head group with twelve component members (Figure 2) diverges considerably from that of phage p2. Remarkably, HHpred searches indicate that the RBP head domain of the P335 phage Tuc2009, whose 3D structure differs significantly from that of phage p2 [19], is the closest predicted structure for these phage RBP head domains. This HHpred result is supported by a lack of sequence alignment between the head domains of phages p2 and Tuc2009 (Supplementary Figure S1B). The saccharide binding site of this domain has not yet been identified. The final and fifth RBP-head group, designated the T4 gp12 group (Figure 2), was also found to diverge considerably from the phage p2 RBP-head group. A HHpred search returned a unique hit, with 100% probability, to the head domain of the gp12 protein from phage T4, representing the T4 RBP located at the extremity of the short fibers that provide irreversible attachment to the host [34]. Furthermore, sequence alignment of these RBPs is consistent with the HHpred result (Supplementary Figure S1C).

Of note, three genomes of the analyzed 936 phage collection, i.e., those of Phi4.2, Phi4R15L and Phi4R16L, were shown to possess two adjacent *rbp* genes, designated *rbp1* and *rbp2*, which have previously been characterized [9]. The *rbp2* gene encodes a ‘traditional’ RBP, while *rbp1* encodes an RBP whose neck domain is on average ~90 residues longer compared to other neck domains, while it also does not resemble any of the five RBP-head groups described above (Figure 2). Therefore, it is likely that these RBPs encode CBMs with novel folds.

### 3.3. RBP Group vs. CWPS Type

It is believed that phages of the 936 group initiate infection of their host by binding to a saccharidic surface receptor on the bacterial cell wall known as the Cell Wall PolySaccharide (CWPS) [14]. It has previously been demonstrated that strains of *L. lactis* can be divided into at least three types (i.e., types A, B, and C, with five C subtypes also identified) based on the genetic composition of the gene cluster responsible for CWPS synthesis [14,15]. The RBPs of the 936 phages have previously been categorized into five groups (I–V) based on sequence similarity and host CWPS preference [14,16], and the groupings defined in the current study are consistent with the previously defined groups (Supplementary Table S1). For example, the p2 RBP group corresponds to RBP group I which exhibits an infection preference for CWPS C strains; the bIL170 RBP group corresponds to RBP group II, members of which recognize strains possessing CWPS B; the T4 gp12 like RBPs are equivalent to RBP group III and recognize strains possessing CWPS type B; the p2-like RBPs correspond to RBP group IV, which infect strains of unknown CWPS types, i.e., the genotypes are undefined using the current multiplex PCR system; and the Tuc2009-like RBPs correspond to the RBP group V phage RBPs that recognize CWPS type A. Interestingly, the host for the P335 group phage Tuc2009 (UC509.9) is a CWPS A strain, which is also the host CWPS-type of the Tuc2009-like RBP phages. Furthermore, p2 and other closely related phages have been shown in this, and previous studies, to infect the lactococcal strain NZ9000, which is a CWPS C strain while bIL170 infects IL1403, a CWPS B strain indicating that the structural and functional data are in agreement, and thus substantiating the genomic and structural groupings.

**Figure 3:**
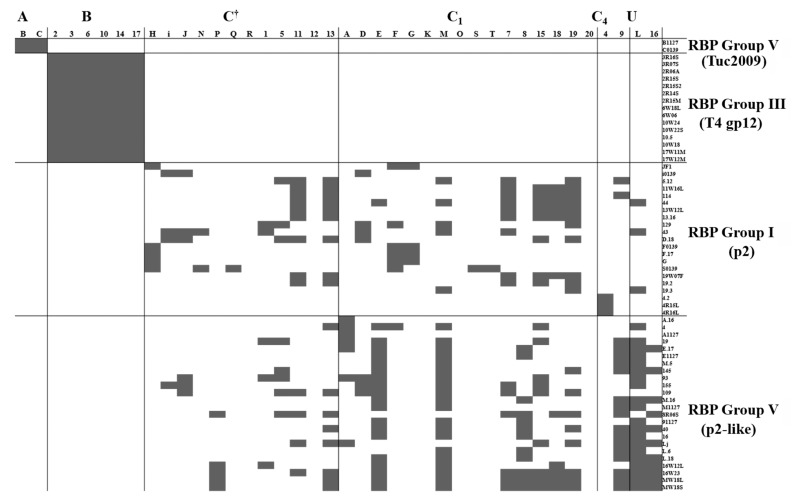
Host ranges of various phages isolated from whey samples between 2009 and 2015 [9,16,20]. Phages are divided by their RBP Group (groups on the right), and host strains by their CWPS Type (types across the top of the table; †denotes C type strains of undetermined subtype, U represents strains of an undetermined CWPS).

To further corroborate the link between RBP-head group of a given phage and CWPS type of its corresponding host, the host range of sixty two 936 group phages isolated from Dutch dairy facilities [16,17,20] was determined against a panel of 40 lactococcal indicator strains. To facilitate analysis of the correlation between RBP group and host CWPS type, the CWPS types of the forty host strains were first determined via multiplex PCR. The results of the host range assays indicate that there is indeed a very strict correlation between the RBP-head group of the phages and the CWPS types of their hosts (Figure 3). For example, Tuc2009-like RBP-head group, or RBP Group V phages, were shown to only infect CWPS type A strains, Phage T4 gp12 RBP-head group/ RBP Group III phages were only able to infect CWPS type B strains, while p2 and p2-like RBP-head group (RBP Groups I and IV) phages infected primarily C type CWPS strains, although without showing preference for particular subtypes.

### 3.4. Bioinformatic Analysis of the Major Tail Proteins

The MTP sequences of the examined 936 phages belong to three main groups, 83 of which harbor an elongated sequence as detailed below. The first group with 30 members is characterized by a ‘short’ MTP of ~215 residues, as revealed by sequence alignments (Supplementary Figure S2, red zone bottom). The MTPs of this group are comparable (but slightly longer) to the short and ‘naked’ MTP forming the tail ring of phage TP901-1 [35]. MTPs of a second group are ~100 residues longer in comparison to those of the first group (Supplementary Figure S2, red and blue zones, top). This is the case for the MTP of phage p2, for which EM structural analysis of the complete virion [32] revealed the presence of a protruding decoration all along the tail (Figure 1D). In the third group, due to a programmed frameshift encoding the TpeX, (a portion of the produced) MTPs are significantly longer than in the two other groups, with lengths of between ~470 and 530 residues (Supplementary Figure S2, red, blue and black zones, middle).

The first ~165 residues of the MTP are highly conserved amongst all three MTP groups, and appear to be structurally unique based on HHpred analysis. This region presumably corresponds to the MTP domain that forms a hexameric ring. Analogous and known MTP structures, i.e., those of phage Lambda [36], T4 [37], T5 [38] possess sequences that are too divergent from those of the 936 phages, and therefore fail to result in structural hits for this region via HHpred analysis (Supplementary Figure S3).

Concerning residues ~165 to 300 of the second and third MTP groups, this domain likely represents an adhesin with an immunoglobulin fold as previously reported for lactococcal phage p2 [32] and the mycobacterial phage Araucaria [39]. The X-ray structure of such a domain has been reported for phage Lambda [40,41]. A similar extension has also been reported for phage SPP1, where the extension is the result of a programmed frameshift [42]. It is worth noting that the frameshift leading to this extension is localized at a different position in the extended MTP of phage SPP1 in comparison to those of the 936 group. In phage SPP1, the frameshift is located just after the MTP hexamer, yielding the ~100 amino acid adhesin domain, while in the 936 group, the frameshift occurs subsequent to the adhesin domain to generate a longer extension [16].

**Figure 4:**
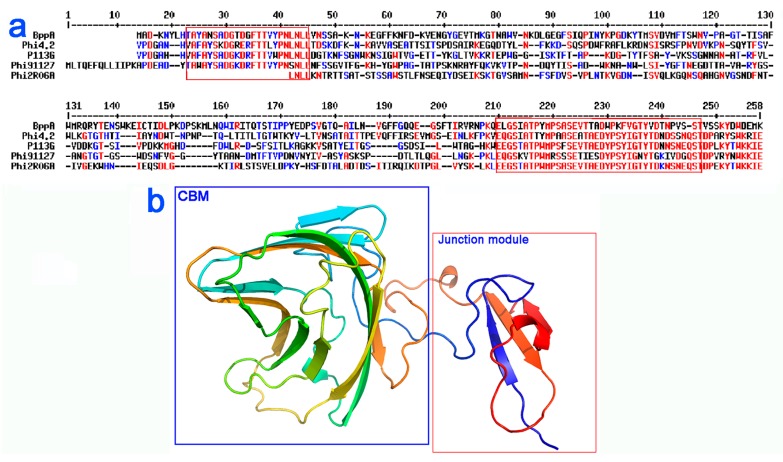
Sequence alignment and predicted structure for the tail protein extensions (TpeX) of 936 group phages. (**a**) Sequence alignment of the TpeX of representative phages highlighting homology to the BppA domain of Tuc2009. The conserved amino acids are in red and the partially conserved in blue. Otherwise in black. Performed with Multalin. (**b**) Structure of BppA CBM and junction domains, which is predicted to be highly similar to the structure of the TpeX. The CBM domain is highlighted in blue, while the junction region, which attaches the CBM domain to the tail tube, is highlighted in red, with the corresponding amino acid sequence also highlighted in (**a**).

This resulting longer extension, known as the TpeX (tail protein extension) [16], is much less conserved than the first 300 amino acids, with the exception of its distal ~50 residues. The long TpeX of the third MTP group can be divided into four sub-groups, which can be distinguished via representative sequences from phages P113G, Phi4.2, Phi91127 and Phi2R06A (Table 2, Supplementary Figure S4). Interestingly, HHpred analysis revealed that all extensions belong to, and are variations of, the Tuc2009 BppA structure [19] (Figure 4a). From the alignment, however, it is apparent that a stretch of residues corresponding to the BppA C-terminus is much more conserved (65–70% identity). In BppA, these residues, together with the *N*-terminal domain, form a small linker between the titin-like domain (which harbors the extension linking BppA to the upper baseplate protein, or BppU) and the CBM domain, anchoring the CBM to the baseplate. To ensure that this high identity stretch had not biased the HHpred output, resulting in BppA producing the top hit at the expense of other matches, a HHpred search was performed following deletion of the N- and C-termini of the TpeX of P113G. The output of this search confirmed BppA as the top hit, indicating that the initial results were not biased, and that the TpeX does, in fact, encode a CBM domain (Supplementary Figure S5).

### 3.5. Bioinformatic Analysis of the Neck Passage Structure Proteins

About two thirds of the assessed phages from the 936 group (73 of 113 analysed in this study) possess an NPS, ranging in size from ~500 to ~900 residues (Table 1 and Supplementary Table S1). Sequence alignment reveals that the first ~180 residues are nearly identical for all members, while the remaining sequences represent seven groups (Table 3; Supplementary Figure S6). HHpred analysis of the N-terminus indicated that the ~130 first residues share the same fold as the *N*-terminal β-sandwich domain of TP901-1 BppU (PDB IDs 4V96, 3UH8), which is a trimeric protein [7]. The TP901-1 BppU is known to assemble as a hexamer of trimers forming a large 18-mer ring around and above the Dit hexameric ring. In this configuration, the 18 BppU proteins are incorporated as six helical bundles, each consisting of three helices, which emerge radially at the periphery of the 18-mer ring. After a kink, they abut to a β-sandwich domain attaching the RBPs to the rest of the baseplate [7,19]. In view of the HHpred output for the NPS, we suggest that a similar 18-mer NPS ring structure forms the collar at the level of tail-capsid connector.

The next structure identified by HHpred in the NPS of some phages (e.g., Phi155) is similar to the three helix bundle of the tail needle protein gp26 of phage P22 [43] (PDB 4lin). Additionally, hits against various carbohydrate binding proteins were obtained. For three NPS groups, represented by phages PhiA16, Phi155 and PhiB1127, HHpred reported the Tuc2009 BppA connector and CBM as the highest hit (Table 3). Sequence alignments of members of these three groups showed that PhiA16 and Phi155 possess CBM domains of strong sequence similarity, while that of PhiB1127 is more distantly related. Noteworthy, for all members of these three groups, the last ~50 residues, which do not form part of the CBM domain, are very close in sequence but remain unassigned by HHpred, possibly indicating a linker domain similar to that observed in the C-terminus of the MTP extensions. In two other NPS groups, represented by phages Phi19.3 and ASCC365, the C-terminal domain possesses a very different fold, as reported by HHpred (Table 3). This domain folds as a β-helix and is present in several glycan hydrolysing enzymes or CBMs. The two remaining NPS groups (represented by Phi114 and ASCC191) exhibit a C-terminal domain with yet another fold, which exhibits structural similarity to the CBM module of carbohydrate esterases [44] (Table 3).

The data analysis permitted the development of a model for the NPS collar, fibres, and CBM modules in which the collar is formed by the ring of the 6 × 3 *N*-terminal β-sandwich domains of BppU from which six helical, trimeric coiled-coil trimers emerge, terminating in 6 × 3 CBMs (Figure 1d–f).

HHpred analysis of a small number of lactococcal phages outside the 936 group revealed that this structure may be widespread in the siphophage world. Indeed, the structure and function of the TP901-1 NPS, a member of the P335 group of lactococcal phages, has been deciphered previously [33]. The TP901-1 NPS is a 72 kDa protein of 667 amino acids. The NPS collar, which is non-essential for infection, was reported to be expressed with the capsid and is proposed to be attached to the portal. This report also provides insightful EM views of the NPS, revealing the size of the collar, the length of the fibers, and the presence of globular structures at the end of the fibres. The finding of similar structural domains in the 936 group phages implies that this phage group employs similar mechanisms of host attachment.

HHpred analysis of NPS_TP901-1_ predicts the same features as those observed for NPS proteins of the 936 family: the *N*-terminal β-sandwich domain of BppU (128 residues) is followed by a helical, trimeric coiled-coil (needle protein GP26 of P22; residues 130-383), and terminates in the BppA adaptor and CBM (Supplementary Figure S7). The dimension of the TP901-1 collar, measured at 160 ± 20 Å, is clearly within the size range of the BppU 18-mer ring (150 Å), while the length of the fibre, measured at 330 ± 40 Å, fits well with the length of a 253 aa-long trimeric coiled-coil (360 Å). Finally, the radius of the globular structures measured 80 ± 15 Å, and the radius of a model of BppA trimer is measured at ~80 Å. Hence, the combination of HHpred reports and structure dimensions fit well with the EM-based estimates.

### 3.6. Fluorescent Binding Assays of NPS-CBM and MTP-TpeX Domains

**Figure 5:**
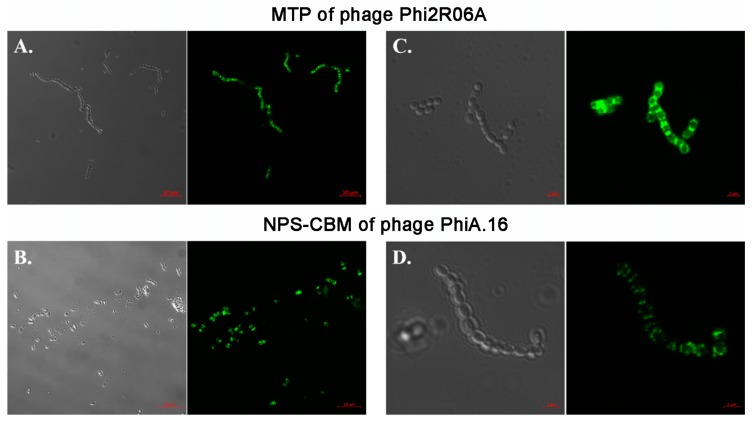
Binding assays of GFP-labelled MTP of Phi2R06A (**A** + **C**) and NPS-CBM of PhiA.16 (**B** + **D**). (**A**) Binding of GFP-labelled TpeX of the MTP of Phi2R06A to host strain *L. lactis* 2. Protein was added at a quantity of 25 μg. (**B**) Binding of the GFP-labelled NPS CBM domain of PhiA.16 to host strain *L. lactis* A. Protein was added at a quantity of 100 μg. (**C**) Binding of the GFP-labelled TpeX of the MTP of Phi2R06A to host strain *L. lactis* 2 at higher resolution, highlighting the localized nature of binding on the cell. Protein was added at a quantity of 25 μg. (**D**) Binding of GFP-labelled NPS CBM domain of PhiA.16 to host strain *L. lactis* A at higher resolution, highlighting the localized nature of binding on the cell. Protein was added at a quantity of 100 μg. Cells were visualized using differential interference contrast (DIC) microscopy (panels on the left), and fluorescent confocal microscopy (panels on the right) at the GFP excitation wavelength of 488 nm. Scale bars correspond to 10 μm in (**A**) and (**B**), and 2 μm in (**C**) and (**D**). Negative controls are provided in Figure 6 (MTP) and in Supplementary Figure S8 (NPS).

In order to validate the predictions of the bioinformatic analyses of the putative MTP-TpeX and NPS CBMs, we decided to explore the CBM/host association in vivo by incubating fluorescently labelled TpeX and NPS proteins or protein domains with host cells and non-host cells, followed by fluorescence microscopy to determine the specific host-binding capabilities of the proteins and their component domains. For this purpose, we expressed the NPS CBM domain of PhiA.16 and the MTP TpeX extension of Phi2R06A. These two proteins were fused to a GFP fluorescent tag at the N-terminus and, after expression and purification, were employed in preliminary binding assays using a fluorescent microscope, followed by confocal binding assays. The micrographs produced highlight the binding of TpeX and NPS domains to host cells (Figure 5).

A concentration of 4.28 µM of the TpeX of Phi2R06A was required for efficient binding (Figure 5A). The CBM domain of the NPS of PhiA.16 was shown to exhibit weaker affinity, requiring a concentration of 9.47 µM (Figure 5B). Even at this high level, the binding efficiency for the NPS-CBM was low, with only a proportion of cells exhibiting visible binding. Since these are ancillary binding domains, it is perhaps unsurprising that the binding affinities differ between the proteins. Furthermore, protein-saccharide interactions may be weak and require higher concentrations of protein in artificial assays to demonstrate such binding. The CBM-containing TpeX and NPS proteins appear to perform localized binding to the host cell surface (as was previously observed in the case of the Dit proteins [17]). With these proteins, binding appeared to favor the polar ends of the cell, where cell division and septum formation occur (Figure 5C,D).

### 3.7. Strain and CWPS Specificity

**Figure 6:**
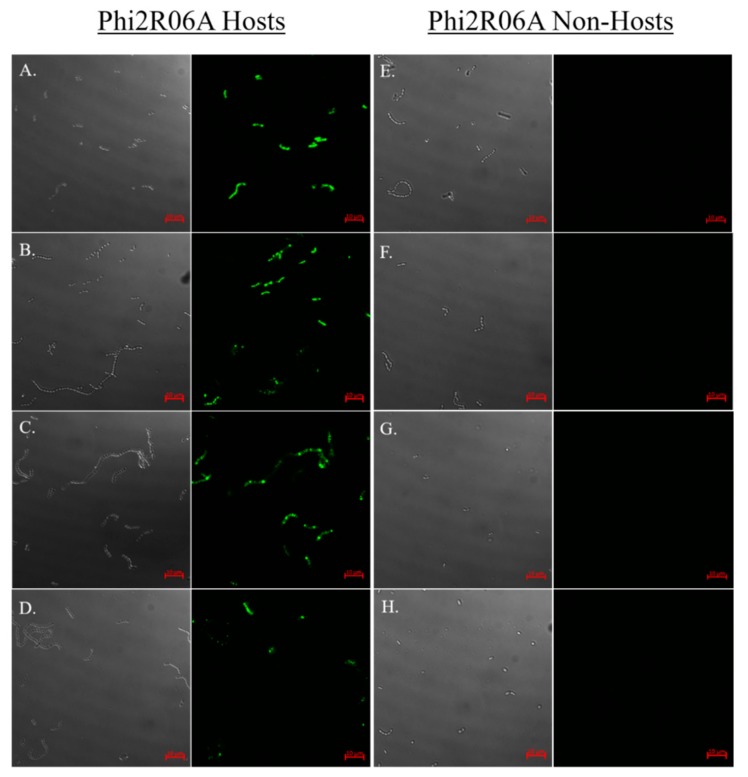
Host binding assay of GFP-labelled TpeX of the MTP of Phi2R06A against eight strains: four hosts which the mature phage is capable of infecting (**A**–**D**), and four non-host strains (**E**–**H**). Binding to host strain *L. lactis* 2 (CWPS Type B; panel **A**), host strain *L. lactis* 10 (CWPS Type B; panel **B**), host strain *L. lactis* 17 (CWPS Type B; panel **C**), host strain *L. lactis* 3 (CWPS Type Unknown; panel **D**), non-host strain *L. lactis* C (CWPS Type A; panel **E**), non-host strain *L. lactis* 4 (CWPS Type C; panel **F**), non-host strain *L. lactis* 13 (CWPS Type C; panel **G**), non-host strain *L. lactis* A (CWPS Type Unknown, panel **H**). MTP was added at a quantity of 25 μg. Cells were visualised using differential interference contrast (DIC) microscopy (panels on the left), and fluorescent confocal microscopy (panels on the right) at wavelength of 488 nm. Scale bars correspond to 10 μm.

To determine if binding of the TpeX CBM is host specific, as has previously proven to be the case for the RBP and Dit [17], the binding of the TpeX of Phi2R06A to four host strains and four non-host strains was examined (Figure 6). The results indicate that the TpeX CBM domain binds to all infection positive hosts, and to none of the non-host strains, exhibiting host-specific binding. Interestingly, with Phi2R14S and Phi10.5 (which exhibit the same infectious range as phage Phi2R06A, see Figure 3), the Dit proteins have previously been shown to exhibit the same binding preference as the MTP of phage Phi2R06A [17]. The poor binding qualities of the NPS-CBM of PhiA.16 and the prohibitive amounts of protein required for successful binding, along with the specific host-range of this phage (Figure 3) prevented host-range analysis of similar depth, although binding was examined on *L. lactis* C (CWPS Type A), *L. lactis* 10 (CWPS Type B), and *L. lactis* E (an additional CWPS Type C strain), with all proving negative for binding.

### 3.8. The Multiplicity of CBM Modules

CBM modules can decorate the Dit [17], MTP, and NPS proteins of 936 phages (the RBP is not counted as a decoration). This leads to the question as to whether these decorations all occur together, or if one kind of decoration is sufficient to ensure host contact is established. The data in Table 1 highlights that only four phages are totally devoid of any such decoration: 936, p2, jj50, and sk1. The majority, 72 of 113, possess an NPS; however, these phages rarely possess an additional decoration besides this NPS, with only two possessing an MTP decoration, and five of them a Dit decoration. It seems, therefore, that NPS decorations generally exclude other decorations (perhaps because they do not bestow additional ecological benefit). In contrast, Dit decorations are often associated with MTP TpeX decorations (20 occurrences out of 30). Furthermore, only one phage within our collection possesses all three different CBM decorations (phage MP1) in addition to its RBP.

Previous examination of Dit extensions revealed four classes of “evolved” Dit, with these classes correlating with the RBP group of the phage [17]. Similar analysis of the MTP- and NPS-associated CBMs presented in this study followed a similar pattern, although perhaps not as highly conservative (Supplementary Table S1). For example, phages possessing p2-like RBPs can be divided into two subsets based on alignments (Figure 2). Comparison of their CBM complement highlights that the smaller subset all possess a Group A NPS while none possess an “evolved” Dit or MTP extension. Conversely, the vast majority of the larger subset (18 of 21 phages) possess an extended Dit and a Subgroup III MTP extension (which is present solely in p2-like RBP phages). Meanwhile, the majority of phages of the p2 RBP group solely possess an NPS (42 of 53), although no correlation is observed between the RBP group and the class of NPS possessed. Finally, phages that encode a Tuc2009-like RBP-head group are very likely to possess a Group G NPS structure, with 10 of 12 phages possessing this alone. Thus, phages appear to incorporate a variety of these CBMs throughout their structure (Figure 1), but in apparent specific combinations that may maximize host binding.

## 4. Discussion

It is now apparent that most members of the 936 group of bacteriophages harbor, in addition to their RBP, one or more CBM-encompassing ‘decorations’ on various structural components, such as the Dit, MTP, and NPS proteins. Indeed, so-called “evolved” Dit proteins, which possess an internal extension encoding a BppA-like CBM, were recently shown to promote binding to the host in many 936 phages [17], as well as certain *Lactobacillus* phages [31,45]. The current study, through a combination of HHpred analysis and experimental verification, suggests that both the MTP extension (also known as the Tail protein extension, or TpeX), and the NPS of 936 phages incorporate CBM domains involved in host binding.

MTP decorations have been studied in the case of phages Lambda [40,41] and SPP1 [42]. However, these were “short” MTP decorations encompassing only the adhesin domain, similar to that of phage p2, while the 936 group MTP TpeX extensions harbor an additional large CBM domain. From this study, it appears that TpeX CBMs may bind to the host with a lower affinity to that previously observed for the “evolved” Dits [17]. We also found that the CBM domain of an NPS demonstrates host-binding activities, although with an apparent low affinity as compared to those of Dit and MTP proteins. Also worth mentioning when discussing the binding capabilities of the CBMs in their monomeric form as analyzed in this study, is the fact that these Dit, MTP, and NPS domains are expected to possess an astounding level of avidity amplification. The multiplicity of the NPS is thought to be 18 (3 × 6), and that of the Dit 12 (2 × 6 as there are two Dit hexamers per baseplate in the 936 group [8]). The multiplicity of tail-associated CBMs is difficult to evaluate since it depends on a programmed frameshift [16], but could reach a theoretical maximum of ~180 modules (6 × 30 rings).

Experiments with different host and non-host strains demonstrated that the various binding modules of the RBP and Dit (both examined previously [17]) and the MTP TpeX extensions (NPS was not tested due to its poor binding capabilites) of a given phage bind strictly to host strains. Therefore, it is likely that all CBMs are binding to the same host CWPS, although perhaps at different component sugars to avoid competition with the RBP. Thus, the combined results of this study and the previous Dit-based study suggest that a two-step infection process, proposed as a classical phage–host binding mechanism, may not occur, although this is currently conjecture. With a two-step mechanism, the ancilliary CBMs perhaps “scan” for suitable hosts via reversible binding, before irreversible binding of the RBP occurs. It now appears more likely that the 936 family CBMs are just as host-specific as the RBP, and simply facilitate increased binding affinity for the phage host, by involving different virion parts and thus allowing flexible binding orientations. Furthermore, it appears that these modules bind most specifically to the cellular growth regions, so as to ensure that a phage preferentially attacks an actively growing bacterium, which will thus be in a metabolic state that is more likely to produce a high number of progeny phages. The septal regions of the cells may present zones of higher concentrations of the sacchardic receptors while it may also be that the integrity of these moieties is higher as they are newly synthesized.

Indeed, the targeted specificity of these CBMs was further apparent in the host ranges of phages isolated from Dutch dairy facilities examined in this study. Phages rigidly adhered to specific infection patterns, infecting only host strains of the CWPS type for which a relationship with their RBP group had already been established [14,16]. This indicates that the phages which are prevelant in these facilities (other phages of the 936 group have previously exhibited the ability to infect two CWPS types, although with a preference for the primary host CWPS type of their RBP group [14]) have evolved to specifically target certain sections of the bacterial population of the starter cultures, likely allowing them to fit into specific niches during the fermentation process. Furthermore, when combining these rigid host ranges with the defined combinations in which the ancilliary CBM domains are coupled with specific RBP types, it may be that phages evolved to incorporate the CBMs in a specific manner, in order to best enhance the binding capabilites of the phage for specific host types. However, due to the fact that large numbers of the available 936 group phage sequences originate from a small number of industrial facilities, it also cannot be precluded that much of the correlation results from the possible evolution of these phages from a limited number of lineages.

Pan-936 group HHpred analysis of RBP proteins has revealed that these phages incorporate a range of structures in the head domain, which is responsible for the RBP’s host binding capabilities [10]. Fascinatingly, 936 group phages appear to incorporate similar RBP head structures to those observed in other lactococcal phage groups, such as P335 phages, and even structures homologous to that of the *E. coli* phage T4. Similarly, structural homologs to the BppA protein of Tuc2009 were identified throughout the tail structure of 936 phages, being encoded by the extended Dits, the MTP TpeX extensions, and NPS proteins. Thus, it appears that phages from widely disparate hosts utilise similar, evolutionarily refined host binding machinery. The structural assembly of CBM decorations of a virion therefore appears to be based on common structural bricks, such as the MTP, Dit, Tal, NPS, or capsid, on which other bricks with functional capacity are assembled in order to achieve maximum infective efficacy under natural conditions. The current findings, in combination with previous work [17,31,45], have elucidated the conservation of CBMs and their functionality and suggest that shuffling of such domains maximizes the binding capabilities of the phage.

## Figures and Tables

**Table 1:** List of the 113 phages from the 936 under scrutiny, along with the types of ancillary CBM domains observed in each.

Phage	RBP Head	Dit ^†^ Class	NPS Group	TpeX Group	Phage	RBP Head	Dit ^†^ Class	NPS Group	TpeX Group
PhiA.16	p2-like		A		ASCC502	p2		E	
PhiLj	p2-like		A		ASCC337	p2		E	
PhiA1127	p2-like		A		ASCC527	p2		E	
Phi19	p2-like		A		ASCC544	p2		E	
Phi4	p2-like		A		ASCC368	p2		E	
Phi17	p2-like	1		III	jm2	p2		D	
Phi145	p2-like	1		III	PhiS0139	p2		A	
Phi109	p2-like	1		III	PhiF.17	p2		A	
Phi93	p2-like	1		III	PhiJF1	p2		A	
PhiM.16	p2-like	1	A		PhiF0139	p2		A	
Phi155	p2-like	1	B		fd13	p2		A	
PhiL.18	p2-like	1		III	PhiG	p2		A	
Phi16	p2-like	1		III	jj50	p2			
Phi40	p2-like	1		III	p2	p2			
PhiM1127	p2-like	1		III	sk1	p2			
PhiE1127	p2-like	1		III	JM1	p2		B	
Phi91127	p2-like	1		III	Phi4.2 ^‡^	p2			II
PhiM.5	p2-like	1		III	Phi4R16L ^‡^	p2			II
PhiL.6	p2-like	4		III	Phi4R15L ^‡^	p2			II
Phi8R06S	p2-like	4		III	PhiB1127	Tuc2009		C	I
Phi16W12L	p2-like	1		III	PhiC0139	Tuc2009		C	I
Phi16W23	p2-like	1		III	ASCC281	Tuc2009		G	
PhiMW18L	p2-like	1		III	ASCC358	Tuc2009		G	
PhiMW18S	p2-like	1		III	ASCC365	Tuc2009		G	
MP1	p2-like	1	A	III	ASCC473	Tuc2009		G	
Phi19.3	p2		D		ASCC497	Tuc2009		G	
Phi19W07F	p2		F		ASCC489	Tuc2009		G	
Phi19.2	p2		D		ASCC284	Tuc2009		G	
Phi15	p2		F		ASCC310	Tuc2009		G	
Phi13W11L	p2		D		ASCC356	Tuc2009		G	
Phi114	p2		F		ASCC532	Tuc2009		G	
Phi13.16	p2		D		bIL170	bIL170		A	
Phi44	p2		F		P272	bIL170			I
ASCC395	p2		E		P113G	bIL170			I
ASCC406	p2		E		p680	bIL170		B	
ASCC397	p2		E		p008	bIL170		G	
ASCC476	p2		E		936	bIL170			
ASCC460	p2		E		Phi10.5	T4 gp12	2		
ASCC506	p2		E		Phi2R14S	T4 gp12	2		
712	p2	3		I	Phi3R07S	T4 gp12	2		
ASCC191	p2		E		Phi2R15M	T4 gp12	2		
Phi11W16L	p2		D		Phi2R15S	T4 gp12	2	B	
PhiD.18	p2		C		Phi2R06A	T4 gp12	2		IV
i0139	p2		C		Phi10W22S	T4 gp12	2		
Phi129	p2		C		Phi10W24	T4 gp12	2		
Phi43	p2		C		Phi17W12M	T4 gp12	2	B	
CB14	p2		E		Phi17W11	T4 gp12	2		
CB19	p2		A		Phi6W06	T4 gp12	2	B	
CB20	p2		A		Phi3R16S	T4 gp12	2		
CB13	p2		E		Phi6W18L	T4 gp12	2		
SL4	p2	3			Phi2R15S2	T4 gp12	2		
Phi5.12	p2			IV	Phi10W18	T4 gp12	2	B	
ASCC273	p2		E		340	T4 gp12		F	
ASCC287	p2		E		645	T4 gp12		F	
ASCC454	p2		E		bIBB29	T4 gp12	2		
Phi7	p2		G	I	P475	T4 gp12			I
ASCC324	p2		E						

^†^ Dit classes as determined previously. ^‡^ These phages possess two receptor binding proteins (RBPs). RBP1, the unique elongated protein, does not fit in to any previously determined RBP. Blank positions on the table indicate where no discernable NPS or TpeX domains were identified, or if the Dit belongs to the classical type.

**Table 2:** TpeX extension subgroups as determined by HHpred analysis.

Subgroup	Lead Phage	HHpred Hit	Corresponding Protein	No. of Phages
I	P113G	5e7t	Tuc2009, BppA	7
II	Phi4.2	5e7t	Tuc2009, BppA	3
III	Phi91127	5e7t	Tuc2009, BppA	18
IV	Phi2R06A	5e7t	Tuc2009, BppA	2

**Table 3:** Neck passage structure (NPS) classes as determined by HHpred analysis.

Group	Lead Phage	HHpred Hit	Corresponding Protein	No. of Phages
Group A	PhiA.16	5e7t	Tuc2009, BppA	16
Group B	Phi155	5e7t	Tuc2009, BppA	7
Group C	PhiB1127	5e7t	Tuc2009, BppA	6
Group D	Phi19.3	1gq8, 2pyg	Pectin	6
Group E	ASCC365	2pyg, 4mxn	Alginate	18
Group F	Phi114	2waa	Carbohydrate esterase	6
Group G	ASCC191	2waa	Carbohydrate esterase	12

## Supplementary Materials

The following are available online at https://www.mdpi.com/1999-4915/11/7/631/s1. Figure S1. Sequence alignment of RBP proteins head domains. Figure S2. Sequence alignment of MTP proteins. Figure S3. HHpred analysis if the core MTP of a representative phage from each of the three major MTP groups. Figure S4. Sequence alignment of the MTP extensions of the 936 group of phages, organised by sub-group. Figure S5. HHpred analysis of the MTP extension of p113G, omitting the N and C termini to prevent bias. Figure S6. Sequence alignment of NPS proteins. Figure S7. HHpred analysis of a NPS from the P335 family lactococcal phage TP901-1. Figure S8. Fluorescent binding assay of the GFP-labelled MTP of p2 to NZ9000. Supplementary Table S1. CBMs of the 936 group of phages. Supplementary Table S2. Bacterial strains used in this study. Supplementary Table S3. Primers used for CWPS typing multiplex PCR and their expected product sizes. Data set S1. DNA sequences of PhiA.16 NPS-CBM and Phi2R06A MTP.

## References

[1] Garneau J.E., Moineau S. (2011). Bacteriophages of lactic acid bacteria and their impact on milk fermentations. Microb. Cell Factories.

[2] Brüssow H. (2018). Population Genomics of Bacteriophages.

[3] Mahony J., Murphy J., van Sinderen D. (2012). Lactococcal 936-type phages and dairy fermentation problems: From detection to evolution and prevention. Front. Microbiol..

[4] Veesler D., Cambillau C. (2011). A common evolutionary origin for tailed-bacteriophage functional modules and bacterial machineries. Microbiol. Mol. Biol. Rev..

[5] Campanacci V., Veesler D., Lichière J., Blangy S., Sciara G., Moineau S., Van Sinderen D., Bron P., Cambillau C. (2010). Solution and electron microscopy characterization of lactococcal phage baseplates expressed in escherichia coli. J. Struct. Biol..

[6] Shepherd D.A., Veesler D., Lichiere J., Ashcroft A.E., Cambillau C. (2011). Unraveling lactococcal phages baseplate assembly by mass spectrometry. Mol. Cell. Proteomics.

[7] Veesler D., Spinelli S., Mahony J., Lichière J., Blangy S., Bricogne G., Legrand P., Ortiz-Lombardia M., Campanacci V., van Sinderen D. (2012). Structure of the phage tp901-1 1.8 mda baseplate suggests an alternative host adhesion mechanism. Proc. Natl. Acad. Sci. USA.

[8] Sciara G., Bebeacua C., Bron P., Tremblay D., Ortiz-Lombardia M., Lichière J., Van Heel M., Campanacci V., Moineau S., Cambillau C. (2010). Structure of lactococcal phage p2 baseplate and its mechanism of activation. Proc. Natl. Acad. Sci. USA.

[9] Hayes S., Duhoo Y., Neve H., Murphy J., Noben J.-P., Franz C., Cambillau C., Mahony J., Nauta A., van Sinderen D. (2018). Identification of dual receptor binding protein systems in lactococcal 936 group phages. Viruses.

[10] Spinelli S., Desmyter A., Verrips C.T., de Haard H.J., Moineau S., Cambillau C. (2006). Lactococcal bacteriophage p2 receptor-binding protein structure suggests a common ancestor gene with bacterial and mammalian viruses. Nat. Struct. Mol. Biol..

[11] Tremblay D.M., Tegoni M., Spinelli S., Campanacci V., Blangy S., Huyghe C., Desmyter A., Labrie S., Moineau S., Cambillau C. (2006). Receptor-binding protein of lactococcus lactis phages: Identification and characterization of the saccharide receptor-binding site. J. Bacteriol..

[12] Ricagno S., Campanacci V., Blangy S., Spinelli S., Tremblay D., Moineau S., Tegoni M., Cambillau C. (2006). Crystal structure of the receptor-binding protein head domain from lactococcus lactis phage bil170. J. Virol..

[13] Spinelli S., Veesler D., Bebeacua C., Cambillau C. (2014). Structures and host-adhesion mechanisms of lactococcal siphophages. Front. Microbiol..

[14] Mahony J., Kot W., Murphy J., Ainsworth S., Neve H., Hansen L.H., Heller K.J., Sørensen S.J., Hammer K., Cambillau C. (2013). Investigation of the relationship between lactococcal host cell wall polysaccharide genotype and 936 phage receptor binding protein phylogeny. Appl. Environ. Microbiol..

[15] Ainsworth S., Sadovskaya I., Vinogradov E., Courtin P., Guerardel Y., Mahony J., Grard T., Cambillau C., Chapot-Chartier M.-P., Van Sinderen D. (2014). Differences in lactococcal cell wall polysaccharide structure are major determining factors in bacteriophage sensitivity. mBio.

[16] Murphy J., Bottacini F., Mahony J., Kelleher P., Neve H., Zomer A., Nauta A., Van Sinderen D. (2016). Comparative genomics and functional analysis of the 936 group of lactococcal siphoviridae phages. Sci. Rep..

[17] Hayes S., Vincentelli R., Mahony J., Nauta A., Ramond L., Lugli G.A., Ventura M., van Sinderen D., Cambillau C. (2018). Functional carbohydrate binding modules identified in evolved dits from siphophages infecting various gram-positive bacteria. Mol. Microbiol..

[18] Collins B., Bebeacua C., Mahony J., Blangy S., Douillard F.P., Veesler D., Cambillau C., van Sinderen D. (2013). Structure and functional analysis of the host-recognition device of lactococcal phage tuc2009. J. Virol..

[19] Legrand P., Collins B., Blangy S., Murphy J., Spinelli S., Gutierrez C., Richet N., Kellenberger C., Desmyter A., Mahony J. (2016). The atomic structure of the phage tuc2009 baseplate tripod suggests that host recognition involves two different carbohydrate binding modules. mBio.

[20] Murphy J., Royer B., Mahony J., Hoyles L., Heller K., Neve H., Bonestroo M., Nauta A., van Sinderen D. (2013). Biodiversity of lactococcal bacteriophages isolated from 3 gouda-type cheese-producing plants. J. Dairy Sci..

[21] Ainsworth S. (2014). Characterisation of Bacteriophage-Host Interactions in Lactococcus Lactis. Ph.D. Thesis.

[22] Lillehaug D. (1997). An improved plaque assay for poor plaque-producing temperate lactococcal bacteriophages. J. Appl. Microbiol..

[23] Hildebrand A., Remmert M., Biegert A., Söding J. (2009). Fast and accurate automatic structure prediction with hhpred. Proteins Struct. Funct. Bioinform..

[24] Söding J., Biegert A., Lupas A.N. (2005). The hhpred interactive server for protein homology detection and structure prediction. Nucleic Acids Res..

[25] Corpet F. (1988). Multiple sequence alignment with hierarchical clustering. Nucleic Acids Res..

[26] Emsley P., Lohkamp B., Scott W.G., Cowtan K. (2010). Features and development of coot. Acta Crystallogr. Sect. D Biol. Crystallogr..

[27] Emsley P., Cowtan K. (2004). Coot: Model-building tools for molecular graphics. Acta Crystallogr. Sect. D Biol. Crystallogr..

[28] Pettersen E.F., Goddard T.D., Huang C.C., Couch G.S., Greenblatt D.M., Meng E.C., Ferrin T.E. (2004). Ucsf chimera—A visualization system for exploratory research and analysis. J. Comput. Chem..

[29] DeLano W.L. (2002). The Pymol Molecular Graphics System. https://pymol.org/2/.

[30] Turchetto J., Sequeira A.F., Ramond L., Peysson F., Brás J.L., Saez N.J., Duhoo Y., Blémont M., Guerreiro C.I., Quinton L. (2017). High-throughput expression of animal venom toxins in escherichia coli to generate a large library of oxidized disulphide-reticulated peptides for drug discovery. Microb. Cell Factories.

[31] Dieterle M.E., Spinelli S., Sadovskaya I., Piuri M., Cambillau C. (2017). Evolved distal tail carbohydrate binding modules of l actobacillus phage j-1: A novel type of anti-receptor widespread among lactic acid bacteria phages. Mol. Microbiol..

[32] Bebeacua C., Tremblay D., Farenc C., Chapot-Chartier M.-P., Sadovskaya I., Van Heel M., Veesler D., Moineau S., Cambillau C. (2013). Structure, adsorption to host, and infection mechanism of virulent lactococcal phage p2. J. Virol..

[33] Vegge C.S., Neve H., Brøndsted L., Heller K.J., Vogensen F.K. (2006). Analysis of the collar-whisker structure of temperate lactococcal bacteriophage tp901-1. Appl. Environ. Microbiol..

[34] Leiman P.G., Shneider M.M., Mesyanzhinov V.V., Rossmann M.G. (2006). Evolution of bacteriophage tails: Structure of t4 gene product 10. J. Mol. Biol..

[35] Bebeacua C., Lai L., Vegge C.S., Brøndsted L., van Heel M., Veesler D., Cambillau C. (2013). Visualizing a complete siphoviridae member by single-particle electron microscopy: The structure of lactococcal phage tp901-1. J. Virol..

[36] Pell L.G., Liu A., Edmonds L., Donaldson L.W., Howell P.L., Davidson A.R. (2009). The x-ray crystal structure of the phage λ tail terminator protein reveals the biologically relevant hexameric ring structure and demonstrates a conserved mechanism of tail termination among diverse long-tailed phages. J. Mol. Biol..

[37] Taylor N.M., Prokhorov N.S., Guerrero-Ferreira R.C., Shneider M.M., Browning C., Goldie K.N., Stahlberg H., Leiman P.G. (2016). Structure of the t4 baseplate and its function in triggering sheath contraction. Nature.

[38] Arnaud C.A., Effantin G., Vivès C., Engilberge S., Bacia M., Boulanger P., Girard E., Schoehn G., Breyton C. (2017). Bacteriophage T5 tail tube structure suggests a trigger mechanism for Siphoviridae DNA ejection. Nat. Commun..

[39] Sassi M., Bebeacua C., Drancourt M., Cambillau C. (2013). The first structure of a mycobacteriophage, araucaria. J. Virol..

[40] Pell L.G., Gasmi-Seabrook G.M., Morais M., Neudecker P., Kanelis V., Bona D., Donaldson L.W., Edwards A.M., Howell P.L., Davidson A.R. (2010). The solution structure of the c-terminal ig-like domain of the bacteriophage λ tail tube protein. J. Mol. Biol..

[41] Fraser J.S., Maxwell K.L., Davidson A.R. (2007). Immunoglobulin-like domains on bacteriophage: Weapons of modest damage?. Curr. Opin. Microbiol..

[42] Auzat I., Dröge A., Weise F., Lurz R., Tavares P. (2008). Origin and function of the two major tail proteins of bacteriophage spp1. Mol. Microbiol..

[43] Bhardwaj A., Casjens S.R., Cingolani G. (2014). Exploring the atomic structure and conformational flexibility of a 320 Å long engineered viral fiber using x-ray crystallography. Acta Crystallogr. Sect. D Biol. Crystallogr..

[44] Montanier C., Money V.A., Pires V.M., Flint J.E., Pinheiro B.A., Goyal A., Prates J.A., Izumi A., Stålbrand H., Morland C. (2009). The active site of a carbohydrate esterase displays divergent catalytic and noncatalytic binding functions. PLoS Biol..

[45] Dieterle M.E., Martin J.F., Durán R., Nemirovsky S.I., Rivas C.S., Bowman C., Russell D., Hatfull G.F., Cambillau C., Piuri M. (2016). Characterization of prophages containing “evolved” dit/tal modules in the genome of lactobacillus casei bl23. Appl. Microbiol. Biotechnol..

