# Supplementary material for: Ubiquitous Carbohydrate Binding Modules Decorate 936 Lactococcal Siphophage Virions
Source: Viruses. 2019 Jul 9;11(7):631. doi: 10.3390/v11070631 (PMC6669499; doi:10.3390/v11070631)
Supplement: Supplementary file 1 [file viruses-11-00631-s001.pdf]

Ubiquitous Carbohydrate Binding Modules Decorate 936 Lactococcal Siphophages Virions

Stephen Hayes[1], Jennifer Mahony[1], Renaud Vincentelli[2,3], Laurie Ramond[2,3,] Arjen Nauta[4], Douwe van Sinderen*[1,5] and Christian Cambillau*[1,2,3]

Corresponding authors: Douwe van Sinderen or Christian Cambillau

Emails: d.vansinderen@ucc.ie or ccambillau@gmail.com

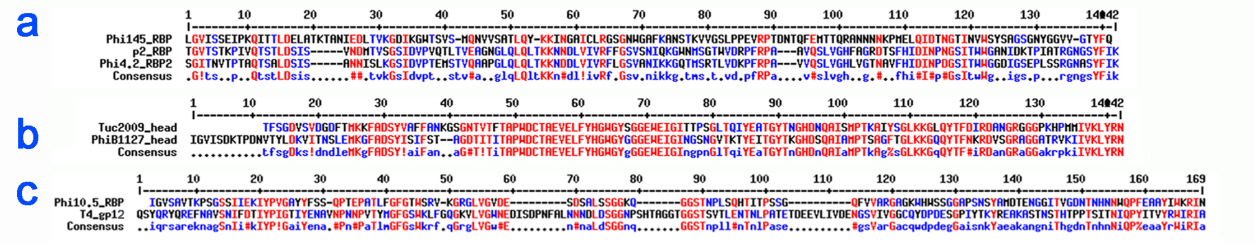

Figure S1. Sequence alignment of RBP proteins head domains. **A.** Sequence alignment of the RBP heads of phages Phi145, p2 and Phi4.2, defining the p2 sub-group. **B.** Sequence alignment of the RBP heads of phages Tuc2009 and PhiB1127, defining the Tuc2009-like group. **C.** Sequence alignment of the RBP head of phages Phi10.5 with T4 gp10, defining the T4 p10-like group. The conserved amino acids are in red and the partially conserved in blue. Otherwise in black. Performed with Multalin.

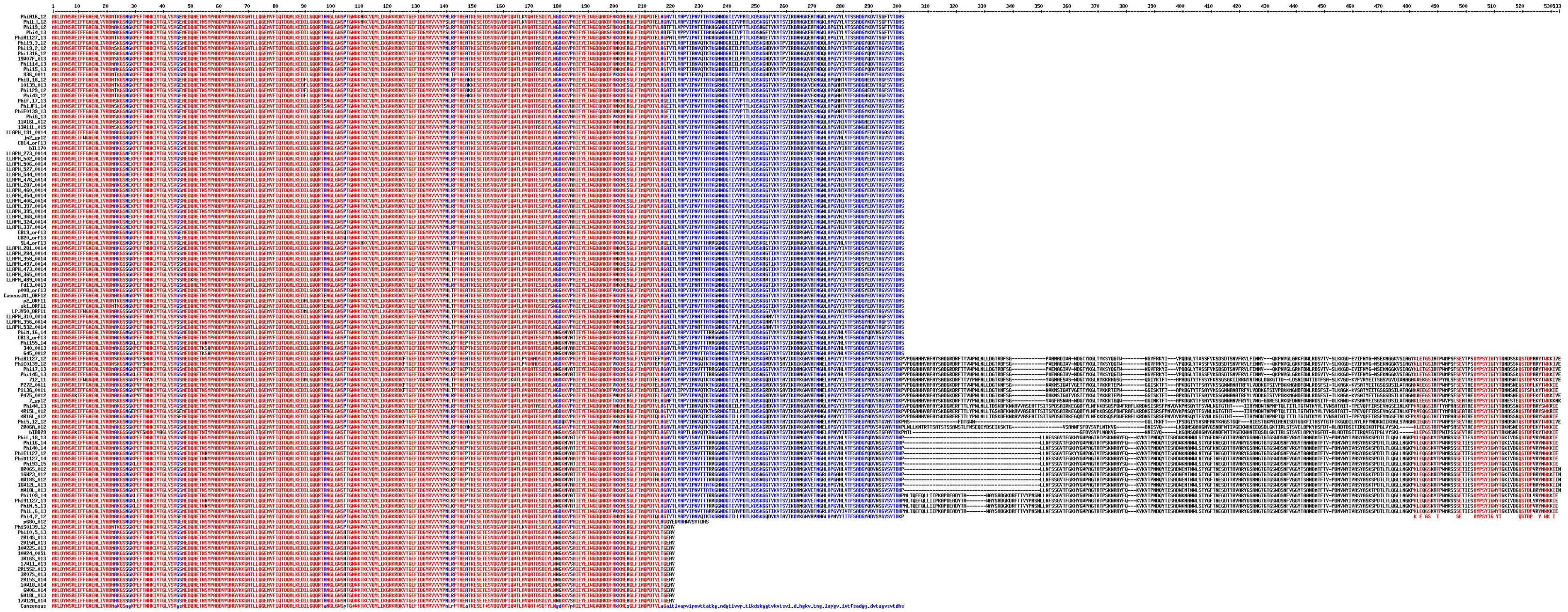

Figure S2. Sequence alignment of MTP proteins. The conserved amino acids are in red and the partially conserved in blue. Otherwise in black. Performed with Multalin [1].

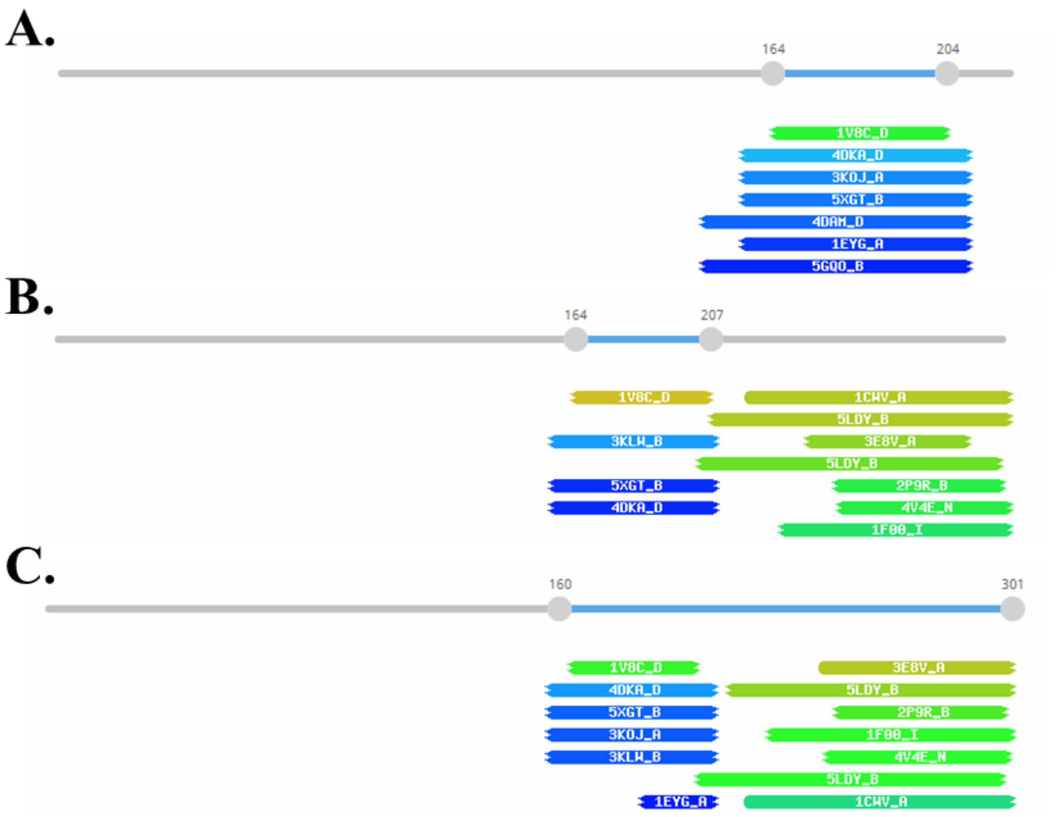

Figure S3. HHpred analysis if the core MTP of a representative phage from each of the three major MTP groups, highlighting the lack of structural hits for the first 160 amino acids. **A.** HHpred analysis of the short MTP of Phi10.5. **B.** HHpred analysis of the MTP of p2, a member of the slightly longer second MTP group. **C.** HHpred analysis of the MTP of PhiC0139, a member of the third MTP group.

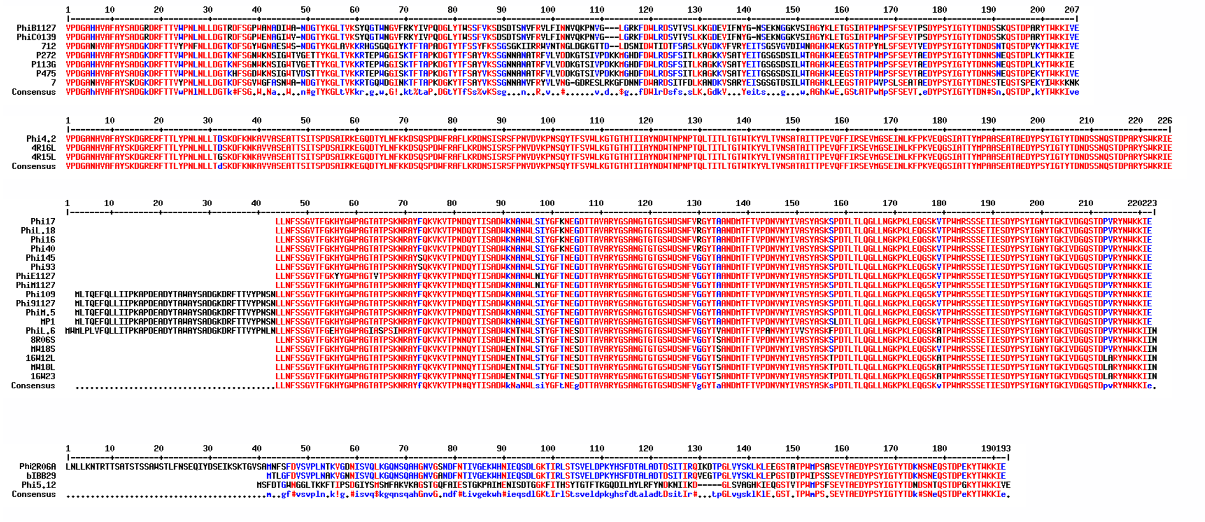

Figure S4. Sequence alignment of the MTP extensions of the 936 group of phages, organised by sub-group. The conserved amino acids are in red and the partially conserved in blue. Otherwise in black. Performed with Multalin [1].

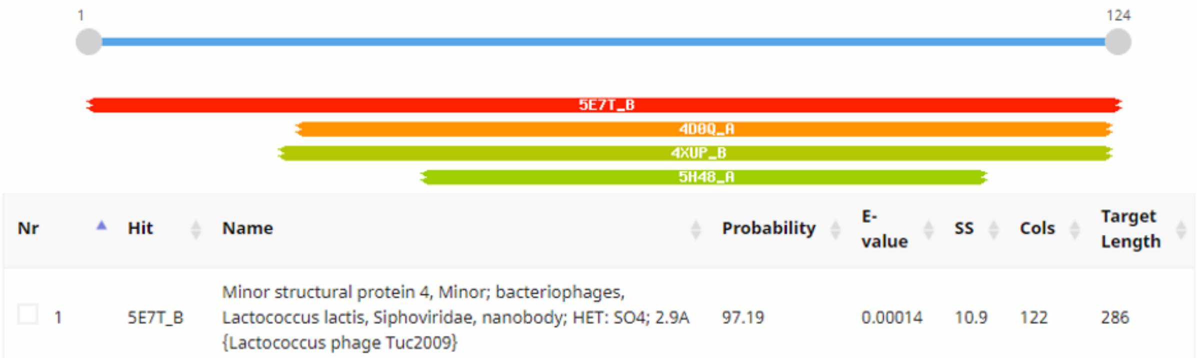

Figure S5. HHpred analysis of the MTP extension of p113G, omitting the N and C termini to prevent bias.

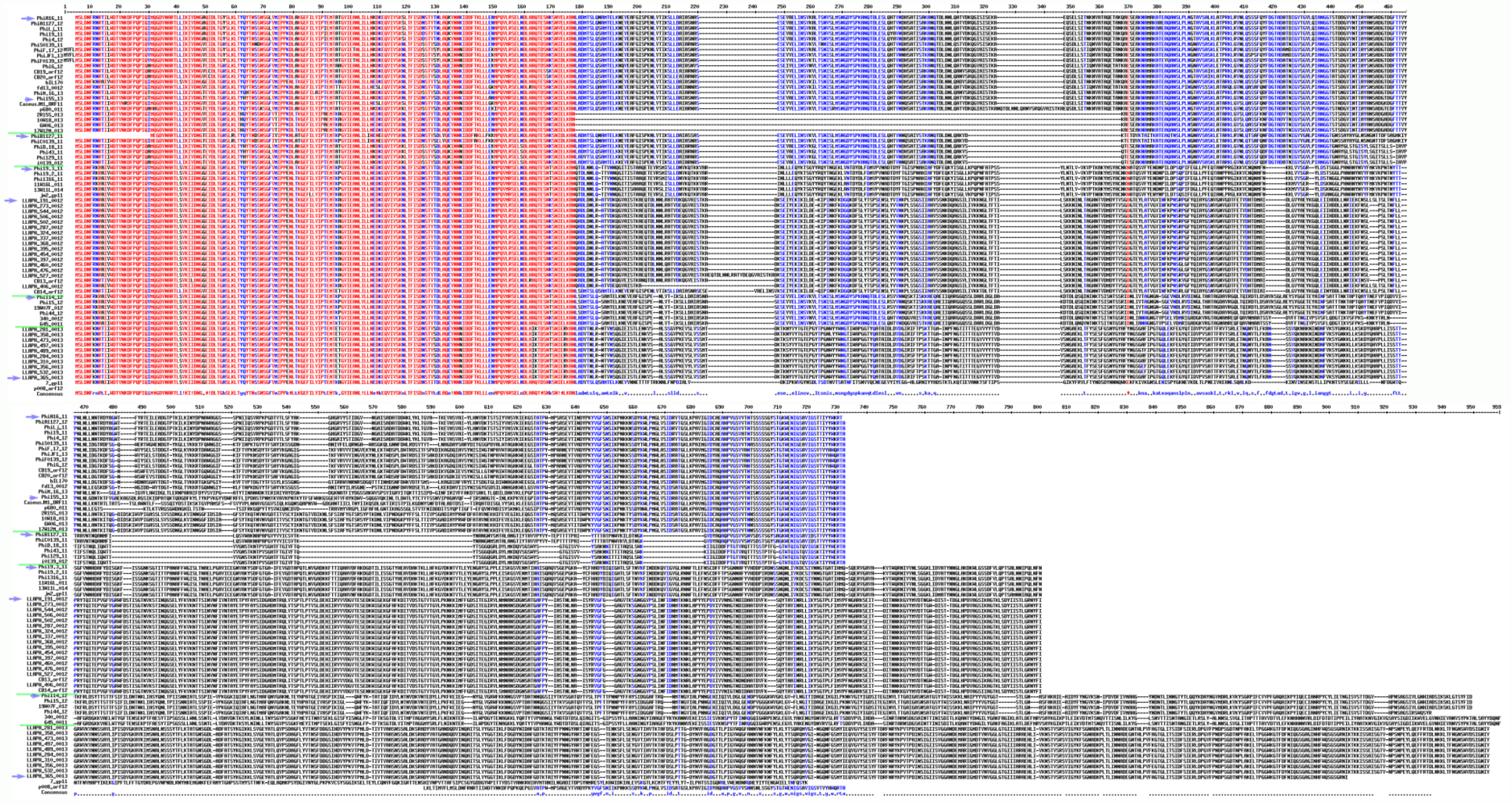

Figure S6. Sequence alignment of NPS proteins. The full-length proteins have been aligned. Fully conserved amino-acids are in red and the partially conserved in blue. Otherwise in black. The phages representing each of the 7 groups distinguished by their C-terminal sequences are identified by a blue arrow. Performed using Multalin [1].

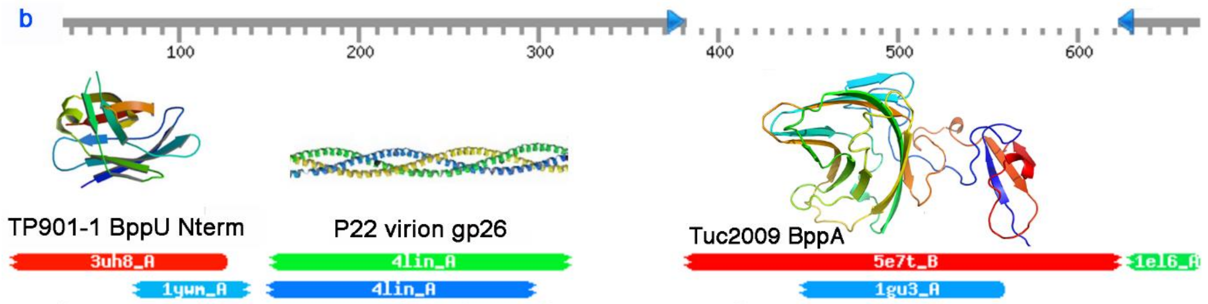

No	Hit		Prob	E-value	P-value	Score	SS	Cols	Query HMM	Template HMM
1	5e7t_B	Minor structural protei	100.0	7.9E-41	2.2E-45	342.4	15.6	218	383-622	2-250 (286)
2	3uh8_A	ORF48; all beta protein	97.7	4.4E-05	1.2E-09	70.4	6.1	120	7-126	2-128 (128)
3	4lin_A	Tail needle protein GP2	67.5	0.68	1.9E-05	48.8	-2.3	142	152-316	63-205 (300)
4	1el6_A	Baseplate structural pr	65.6	4.4	0.00012	41.4	3.1	39	629-667	175-219 (219)
5	1ywm_A	C protein alpha-antigen	55.6	9.7	0.00027	38.0	3.5	61	76-137	71-139 (200)
6	1gu3_A	Endoglucanase C; carboh	53.5	59	0.0016	29.4	8.1	91	447-559	50-149 (149)

Figure S7. HHpred analysis of a NPS from the P335 family lactococcal phage TP901-1. The N-terminus BppU domain, a triple-helix and a BppA module are successively identified.

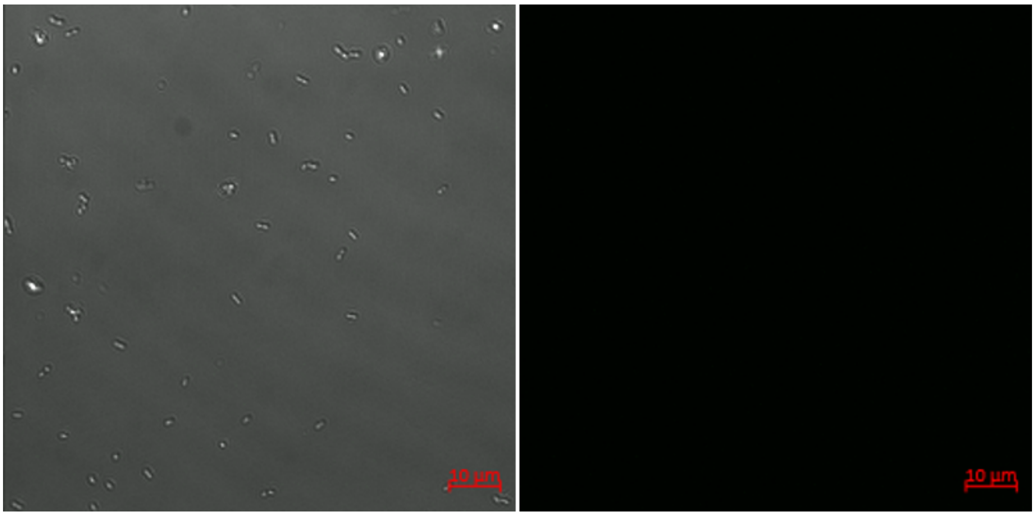

Figure S8. Fluorescent binding assay of the GFP-labelled MTP of p2 to NZ9000. A range of protein quantities between 5 – 100 µg were examined. Cells were visualised using differential interference contrast (DIC) microscopy (panels on the left), and fluorescent confocal microscopy (panels on the right) at excitement wavelengths of 488 nm for GFP-labelled proteins. Scale bars correspond to 10 µm.

Supplementary Table S1. CBMs of the 936 group of phages.

Phage	Accession No.	RBP Head	RBP Group*	Dit†	Size (aa)	NPS	Size (aa)	MTP	Size (aa)
PhiA.16	KP793102	p2-like	Group IV			Group A	651		
PhiLj	KP793133	p2-like	Group IV			Group A	652		
PhiA1127	KP793106	p2-like	Group IV			Group A	652		
Phi19	KP793103	p2-like	Group IV			Group A	652		
Phi4	KP793101	p2-like	Group IV			Group A	652		
Phi17	KP793114	p2-like	Group IV	Class 1	491			Subgroup III	179
Phi145	KM091444	p2-like	Group IV	Class 1	491			Subgroup III	179
Phi109	KP793121	p2-like	Group IV	Class 1	491			Subgroup III	220
Phi93	KM091443	p2-like	Group IV	Class 1	491			Subgroup III	179
PhiM.16	KP793128	p2-like	Group IV	Class 1	491	Group A	654		
Phi155	KP793130	p2-like	Group IV	Class 1	492	Group B	673		
PhiL.18	KP793120	p2-like	Group IV	Class 1	491			Subgroup III	179
Phi16	KP793135	p2-like	Group IV	Class 1	491			Subgroup III	179
Phi40	KP793127	p2-like	Group IV	Class 1	491			Subgroup III	179
PhiM1127	KP793132	p2-like	Group IV	Class 1	492			Subgroup III	179
PhiE1127	KP793131	p2-like	Group IV	Class 1	492			Subgroup III	179
Phi91127	KP793125	p2-like	Group IV	Class 1	491			Subgroup III	220
PhiM.5	KP793126	p2-like	Group IV	Class 1	491			Subgroup III	220
PhiL.6	KP793122	p2-like	Group IV	Class 4	446			Subgroup III	223
Phi8R06S	KX346242	p2-like	Group IV	Class 4	466			Subgroup III	180
Phi16W12L	KX379672	p2-like	Group IV	Class 1	492			Subgroup III	180
Phi16W23	KX346249	p2-like	Group IV	Class 1	492			Subgroup III	180
PhiMW18L	KX379673	p2-like	Group IV	Class 1	492			Subgroup III	180
PhiMW18S	KX346250	p2-like	Group IV	Class 1	492			Subgroup III	180
MP1	MG779474	p2-like	Group IV	Class 1	491	Group A	654	Subgroup III	220
Phi19.3	KP793105	p2	Group I			Group D	728		
Phi19W07F	KX379670	p2	Group I			Group F	862		
Phi19.2	KP793111	p2	Group I			Group D	728		
Phi15	KM091442	p2	Group I			Group F	862		
Phi13W11L	KX379671	p2	Group I			Group D	728		
Phi114	KP793115	p2	Group I			Group F	862		

Phage	Accession No.	RBP Head	RBP Group*	Dit†	Size (aa)	NPS	Size (aa)	MTP	Size (aa)
Phi13.16	KP793116	p2	Group I			Group D	728		
Phi44	KP793124	p2	Group I			Group F	848		
ASCC395	JQ740799	p2	Group I			Group E	723		
ASCC406	JQ740801	p2	Group I			Group E	703		
ASCC397	JQ740800	p2	Group I			Group E	723		
ASCC476	JQ740806	p2	Group I			Group E	723		
ASCC460	JQ740803	p2	Group I			Group E	723		
ASCC506	JQ740810	p2	Group I			Group E	723		
712	DQ227763	p2	Group I	Class 3	471			Subgroup I	201
ASCC191	JQ740787	p2	Group I			Group E	726		
Phi11W16L	KX346246	p2	Group I			Group D	728		
PhiD.18	KP793107	p2	Group I			Group C	512		
i0139	KX379665	p2	Group I			Group C	512		
Phi129	KP793112	p2	Group I			Group C	512		
Phi43	KP793110	p2	Group I			Group C	512		
CB14	FJ848883	p2	Group I			Group E	734		
CB19	FJ848884	p2	Group I			Group A	656		
CB20	FJ848885	p2	Group I			Group A	656		
CB13	FJ848882	p2	Group I			Group E	723		
SL4	FJ848881	p2	Group I	Class 3	468				
Phi5.12	KP793108	p2	Group I					Subgroup IV	149
ASCC273	JQ740788	p2	Group I			Group E	723		
ASCC287	JQ740791	p2	Group I			Group E	723		
ASCC454	JQ740802	p2	Group I			Group E	723		
Phi7	KC182552	p2	Group I			Group G	641	Subgroup I	205
ASCC324	JQ740793	p2	Group I			Group E	723		
ASCC502	JQ740809	p2	Group I			Group E	723		
ASCC337	JQ740794	p2	Group I			Group E	723		
ASCC527	JQ740811	p2	Group I			Group E	746		
ASCC544	JQ740814	p2	Group I			Group E	723		
ASCC368	JQ740798	p2	Group I			Group E	723		
jm2	KC182546	p2	Group I			Group D	728		

Phage	Accession No.	RBP Head	RBP Group*	Dit†	Size (aa)	NPS	Size (aa)	MTP	Size (aa)
PhiS0139	KP793134	p2	Group I			Group A	651		
PhiF.17	P793113	p2	Group I			Group A	660		
PhiJF1	KP793129	p2	Group I			Group A	660		
PhiF0139	KP793118	p2	Group I			Group A	660		
fd13	KC182545	p2	Group I			Group A	650		
PhiG	KP793117	p2	Group I			Group A	656		
jj50	DQ227764	p2	Group I						
p2	GQ979703	p2	Group I						
sk1	AF011378	p2	Group I						
JM1	KC522412	p2	Group I			Group B	466		
Phi4.2 RBP2**	KP793123	p2	Group I					Subgroup II	226
Phi4R16L RBP2**	KX379667	p2	Group I					Subgroup II	226
Phi4R15L RBP2**	KX379668	p2	Group I					Subgroup II	226
PhiB1127	KP793104	Tuc2009-like	Group V			Group C	494	Subgroup I	202
PhiC0139	KP793109	Tuc2009-like	Group V			Group C	520	Subgroup I	202
ASCC281	JQ740789	Tuc2009-like	Group V			Group G	888		
ASCC358	JQ740796	Tuc2009-like	Group V			Group G	888		
ASCC365	JQ740797	Tuc2009-like	Group V			Group G	888		
ASCC473	JQ740805	Tuc2009-like	Group V			Group G	888		
ASCC497	JQ740808	Tuc2009-like	Group V			Group G	888		
ASCC489	JQ740807	Tuc2009-like	Group V			Group G	888		
ASCC284	JQ740790	Tuc2009-like	Group V			Group G	888		
ASCC310	JQ740792	Tuc2009-like	Group V			Group G	888		
ASCC356	JQ740795	Tuc2009-like	Group V			Group G	888		
ASCC532	JQ740812	Tuc2009-like	Group V			Group G	888		
bIL170	AF009630	bIL170	Group II			Group A	653		
P272	KC182549	bIL170	Group II					Subgroup I	206
P113G	KC182548	bIL170	Group II					Subgroup I	206
p680	KC182551	bIL170	Group II			Group B	664		
p008	DQ054536	bIL170	Group II			Group G	145		
936	KC182544	bIL170	Group II						
Phi10.5	KP793119	T4 gp10	Group III	Class 2	486				

Phage	Accession No.	RBP Head	RBP Group*	Dit†	Size (aa)	NPS	Size (aa)	MTP	Size (aa)
Phi2R14S	KX346236	T4 gp12	Group III	Class 2	486				
Phi3R07S	KX346241	T4 gp12	Group III	Class 2	486				
Phi2R15M	KX346237	T4 gp12	Group III	Class 2	486				
Phi2R15S	KX346238	T4 gp12	Group III	Class 2	486	Group B	533		
Phi2R06A	KX346240	T4 gp12	Group III	Class 2	486			Subgroup IV	221
Phi10W22S	KX379669	T4 gp12	Group III	Class 2	486				
Phi10W24	KX346245	T4 gp12	Group III	Class 2	486				
Phi17W12M	KX346248	T4 gp12	Group III	Class 2	486	Group B	533		
Phi17W11	KX346247	T4 gp12	Group III	Class 2	486				
Phi6W06	KX346243	T4 gp12	Group III	Class 2	486	Group B	533		
Phi3R16S	KX379666	T4 gp12	Group III	Class 2	486				
Phi6W18L	KX346244	T4 gp12	Group III	Class 2	486				
Phi2R15S2	KX346239	T4 gp12	Group III	Class 2	486				
Phi10W18	KX379664	T4 gp12	Group III	Class 2	486	Group B	533		
340	KC182542	T4 gp12	Group III			Group F	880		
645	KC182543	T4 gp12	Group III			Group F	880		
bIBB29	EU221285	T4 gp12	Group III	Class 2	486				
P475	KC182550	T4 gp12	Group III					Subgroup I	207

*****As determined previously [2, 3]. †Dit class as determined previously [4]. **These phages possess two RBPs. RBP1, the unique elongated protein, does not fit in to any previously determined RBP group.

Supplementary Table S2. Bacterial strains used in this study.

Bacterial Strain	Source
Lactococcus lactis strains *A* - *T*	[5]
Lactococcus lactis strains 1 - 20	[5]
NZ9000	[6]
IL1403	[7]
ASCC92	[8]
ASCC385	[8]
E8	[9]

Supplementary Table S3. Primers used for CWPS typing multiplex PCR and their expected product sizes.

CWPS Type PCR

CWPS Type	Primer	Sequence (5'-3')	Amplicon Size (bp)
A	UC-CVfw	GTGCCTATGCTCCGTTAGTC	442
	UC-CVrv	CGAGGGCCAATCTCTTTACC	
B	IL-KFfw	GATTCAGTTGCACGGCCG	183
	IL-KFrv	AGTAAGGGGGCGGATTGTG	
C	MG-SKfw	AAAGCTCATCTTTCCCCTGTTGT	686
	MG-SKrv	GCACCATAGTCTGGAATAAGACC	
Control	CONfw	GTACACTATGTTTATAACAATCATCCAG	891
	CONrv	GCAAACCAGATTCAAAGTCAGTATG	

C Type CWPS Subtype PCR

CWPS Type	Primer	Sequence (5'-3')	Amplicon Size (bp)
C_1	C1-fw	GTCATCAAACATACTTTCGTC	650
	C1-rv	AAGTTTTGCCATTGTTTCTCC	
C_2	C2-fw	GAACAATGGATTATTTATGCTGA	450
	C2-rv	ATTCCCATTTTCAGCAACAAG	
C_3	C3-fw	GTTGTAATTGTTACTAGCCAG	250
	C3-rv	TCAATCGCATTATAGATTACACC	
C_4	C4-fw	GATTTTATTCGAGGCTTAGCA	968
	C4-rv	TAGCATTACAATCAATCTGTCA	
C_5	C5-fw	GATTATATTCGGGGCTTAGCA	1141
	C5-rv	TGTAATATGGTATTGTCTAGCA	

Data set 1

PhiA.16-NPS (1440 bp)
ATGGGAGTTAGCAAAGGTGAAGAACTGTTTACGGGCGTTGTGCCGATCCTGGTGGAACTGGACGGTGA
TGTTAATGGTCATAAATTCTCTGTGAGTGGCGAAGGTGAAGGCGATGCGACCTATGGTAAACTGACGC
TGAAATTTATTTGCACCACCGGTAAACTGCCGGTTCCGTGGCCGACCCTGGTCACCACCCTGACCTAC
GGTGTGCAGTGTTTCGCACGCTATCCGGATCATATGAAACAACACGACTTTTTCAAAAGCGCTATGCC
GGAAGGTTACGTTCAGGAACGTACCATTTTCTTTAAAGATGACGGCAACTACAAAACCCGCGCCGAAG
TCAAATTTGAAGGTGATACGCTGGTGAACCGTATTGAACTGAAAGGCATCGATTTCAAAGAAGACGGT
AATATCCTGGGCCATAAACTGGAATACAACTACAACTCACACAAAGTTTACATTACCGCGGATAAACA
GAAAAACGGTATCAAAGTCAACTTCAAAACGCGTCATAACATCGAAGATGGCTCTGTGCAACTGGCCG
ACCACTACCAGCAAAACACCCCGATCGGTGATGGCCCGGTTCTGCTGCCGGACAATCATTATCTGTCC
ACCCAGTCAGCACTGTCGAAAGATCCGAATGAAAAACGCGACCACATGGTGCTGCTGGAATTTGTTAC
CGCGGCCGGTATTACGCTGGGCATGGATGAACTGTACAAAAGCTCTGGTCCATCGGGCAGCAGCCATC
ATCATCATCATCACAGCAGCGGCCCTCAGCaaggGCTGAGGGAAAACCTGTACTTCCAGGGCGATGAC
GGAGTTGTAAATACCATTGCCTATGCCAATAGCGCAGACGGTACTGACGGTTTCACGACTGTTTATCC
TAATTTGAATCTGTTGAATAATACACGTGATTATGCTGGATGGACATTTTATCGCACAGAAATATTAG
AAGCGGATGGAACGCCTACTAAGATTCTTAAAATTAATTACGATCCTAACGCTTGGGCAGGTGGATCT
TCACCCAATATCATTCAGTCAGTAAGACCTAAACCTGGCGATACAGTTACTCTTAGTTTCTATGCAAA
AGGACATGGTAGGGTTTATTCTACTATTGACGGTGTTAATGGAGCAATTAGCGCCGATGCTACTGATG
ATTGGAAGCTTTACAAGTTGACTGGGGTGGCTACGAAAGAAGTTCATAGTGTCGCTATCTATCTACAC
AACGTTGACAAGACATCAACAAGCATTTATGTTCATTCCGTTAAAATAGAAAAAGGCTCAACCGCCAC
CCCTTGGATGCCATCAGCTAGCGAAGTAACAATAAATGACTATCCGAAGTATGTGGGGTTTAGTAATA
GCATTAAACCAAATAAGAAAAATTCTGATTACAAATGGCTACCAATGGGGTTAGTGTCAATTGATAGG
GTTACAGGCTAA

Phi2R06A-MTP (1467 bp)
ATGGGAGTTAGCAAAGGTGAAGAACTGTTTACGGGCGTTGTGCCGATCCTGGTGGAACTGGACGGTGA
TGTTAATGGTCATAAATTCTCTGTGAGTGGCGAAGGTGAAGGCGATGCGACCTATGGTAAACTGACGC
TGAAATTTATTTGCACCACCGGTAAACTGCCGGTTCCGTGGCCGACCCTGGTCACCACCCTGACCTAC
GGTGTGCAGTGTTTCGCACGCTATCCGGATCATATGAAACAACACGACTTTTTCAAAAGCGCTATGCC
GGAAGGTTACGTTCAGGAACGTACCATTTTCTTTAAAGATGACGGCAACTACAAAACCCGCGCCGAAG
TCAAATTTGAAGGTGATACGCTGGTGAACCGTATTGAACTGAAAGGCATCGATTTCAAAGAAGACGGT
AATATCCTGGGCCATAAACTGGAATACAACTACAACTCACACAAAGTTTACATTACCGCGGATAAACA
GAAAAACGGTATCAAAGTCAACTTCAAAACGCGTCATAACATCGAAGATGGCTCTGTGCAACTGGCCG
ACCACTACCAGCAAAACACCCCGATCGGTGATGGCCCGGTTCTGCTGCCGGACAATCATTATCTGTCC
ACCCAGTCAGCACTGTCGAAAGATCCGAATGAAAAACGCGACCACATGGTGCTGCTGGAATTTGTTAC
CGCGGCCGGTATTACGCTGGGCATGGATGAACTGTACAAAAGCTCTGGTCCATCGGGCAGCAGCCATC
ATCATCATCATCACAGCAGCGGCCCTCAGCaaggGCTGAGGGAAAACCTGTACTTCCAGGGCGCGCCC
GACGGGGCTAACCACGTAGCCTTTGCGTATAGCGCAGACGGAAAAGATAGATTCACGACTGTCTATCC
GAATTTGAATTTGTTAAAAAACACGAGAACGACATCGGCAACTTCAACTTCATCAGCTTGGAGTACTT
TATTTAATTCTGAACAAATATATGACTCCGAAATTAAATCTAAAACTGGAGTTTCAGCAATGAACTTT
AGTTTCGATGTTTCTGTACCATTGAATACTAAAGTTGGAGATAATATTTCTGTCCAGCTTAAAGGTCA
AAATTCTCAAGCTCATGGAAATGTTGGATCCAATGATTTCAACACAATTGTTGGTGAAAAGTGGCATA
ATATTGAACAAAGCGATTTAGGTAAAACAATTCGTTTAAGCACTTCAGTGGAATTAGATCCTAAATAT
CATTCTTTTGATACTGCTTTAGCTGATACTGATAGTATTACTATTAGACAAATCAAAGACACGCCAGG
ACTTGTGTATTCTAAATTAAAACTTGAAGAAGGTTCAACCGCAACTCCTTGGATGCCCTCAGCTAGTG
AAGTAACAGCCGAAGATTATCCAAGCTATATAGGAACATATACTGATAAAAACTCCAATGAACAAAGT
ACAGACCCAGAAAAATATACTTGGAAAAAAATAGAATAA

References

1. Corpet, F., Multiple sequence alignment with hierarchical clustering. *Nucleic acids research* **1988,** 16, (22), 10881-10890.
2. Mahony, J.; Kot, W.; Murphy, J.; Ainsworth, S.; Neve, H.; Hansen, L. H.; Heller, K. J.; Sørensen, S. J.; Hammer, K.; Cambillau, C., Investigation of the relationship between lactococcal host cell wall polysaccharide genotype and 936 phage receptor binding protein phylogeny. *Applied and environmental microbiology* **2013**, AEM. 00653-13.
3. Murphy, J.; Bottacini, F.; Mahony, J.; Kelleher, P.; Neve, H.; Zomer, A.; Nauta, A.; van Sinderen, D., Comparative genomics and functional analysis of the 936 group of lactococcal Siphoviridae phages. *Scientific reports* **2016,** 6.
4. Hayes, S.; Vincentelli, R.; Mahony, J.; Nauta, A.; Ramond, L.; Lugli, G. A.; Ventura, M.; van Sinderen, D.; Cambillau, C., Functional Carbohydrate Binding Modules Identified in evolved Dits from Siphophages Infecting various Gram-positive Bacteria. *Molecular microbiology* **2018**.
5. Murphy, J.; Royer, B.; Mahony, J.; Hoyles, L.; Heller, K.; Neve, H.; Bonestroo, M.; Nauta, A.; van Sinderen, D., Biodiversity of lactococcal bacteriophages isolated from 3 Gouda-type cheese-producing plants. *Journal of dairy science* **2013,** 96, (8), 4945-4957.
6. Kuipers, O. P.; de Ruyter, P. G.; Kleerebezem, M.; de Vos, W. M., Quorum sensing-controlled gene expression in lactic acid bacteria. *Journal of Biotechnology* **1998,** 64, (1), 15-21.
7. Bolotin, A.; Wincker, P.; Mauger, S.; Jaillon, O.; Malarme, K.; Weissenbach, J.; Ehrlich, S. D.; Sorokin, A., The complete genome sequence of the lactic acid bacterium Lactococcus lactis ssp. lactis IL1403. *Genome research* **2001,** 11, (5), 731-753.
8. Castro-Nallar, E.; Chen, H.; Gladman, S.; Moore, S. C.; Seemann, T.; Powell, I. B.; Hillier, A.; Crandall, K. A.; Chandry, P. S., Population genomics and phylogeography of an Australian dairy factory derived lytic bacteriophage. *Genome biology and evolution* **2012,** 4, (3), 382-393.
9. Gopal, P. K.; Crow, V. L., Characterization of loosely associated material from the cell surface of Lactococcus lactis subsp. cremoris E8 and its phage-resistant variant strain 398. *Applied and environmental microbiology* **1993,** 59, (10), 3177-3182.
